# Pb-Induced Avoidance-Like Chloroplast Movements in Fronds of *Lemna trisulca* L.

**DOI:** 10.1371/journal.pone.0116757

**Published:** 2015-02-03

**Authors:** Sławomir Samardakiewicz, Weronika Krzeszowiec-Jeleń, Waldemar Bednarski, Artur Jankowski, Szymon Suski, Halina Gabryś, Adam Woźny

**Affiliations:** 1 Laboratory of Electron and Confocal Microscopy, Faculty of Biology, Adam Mickiewicz University, Poznań, Poland; 2 Department of Plant Biotechnology, Faculty of Biochemistry, Biophysics and Biotechnology, Jagiellonian University, Kraków, Poland; 3 Institute of Molecular Physics, Polish Academy of Sciences, Poznań, Poland; 4 Laboratory of General Botany, Institute of Experimental Biology, Faculty of Biology, Adam Mickiewicz University, Poznań, Poland; 5 Laboratory of Electron Microscopy, Nencki Institute of Experimental Biology, Polish Academy of Sciences, Warszawa, Poland; ISA, PORTUGAL

## Abstract

Lead ions are particularly dangerous to the photosynthetic apparatus, but little is known about the effects of trace metals, including Pb, on regulation of chloroplast redistribution. In this study a new effect of lead on chloroplast distribution patterns and movements was demonstrated in mesophyll cells of a small-sized aquatic angiosperm *Lemna trisulca* L. (star duckweed). An analysis of confocal microscopy images of *L. trisulca* fronds treated with lead (15 μM Pb^2+^, 24 h) in darkness or in weak white light revealed an enhanced accumulation of chloroplasts in the profile position along the anticlinal cell walls, in comparison to untreated plants. The rearrangement of chloroplasts in their response to lead ions in darkness was similar to the avoidance response of chloroplasts in plants treated with strong white light. Transmission electron microscopy X-ray microanalysis showed that intracellular chloroplast arrangement was independent of the location of Pb deposits, suggesting that lead causes redistribution of chloroplasts, which looks like a light-induced avoidance response, but is not a real avoidance response to the metal. Furthermore, a similar redistribution of chloroplasts in *L. trisulca* cells in darkness was observed also under the influence of exogenously applied hydrogen peroxide (H_2_O_2_). In addition, we detected an enhanced accumulation of endogenous H_2_O_2_ after treatment of plants with lead. Interestingly, H_2_O_2_-specific scavenger catalase partly abolished the Pb-induced chloroplast response. These results suggest that H_2_O_2_ can be involved in the avoidance-like movement of chloroplasts induced by lead. Analysis of photometric measurements revealed also strong inhibition (but not complete) of blue-light-induced chloroplast movements by lead. This inhibition may result from disturbances in the actin cytoskeleton, as we observed fragmentation and disappearance of actin filaments around chloroplasts. Results of this study show that the mechanisms of the toxic effect of lead on chloroplasts can include disturbances in their movement and distribution pattern.

## Introduction

The movement of organelles makes it possible for cells to maintain homeostasis and adapt to the changing environmental conditions [1,2,3]. In plant cells, movements of chloroplasts play a particularly important role. They may be either autonomous or linked with cytoplasmic streaming [4,5,6]. Light-induced directional movements of chloroplasts are an example of movement independent of cyclosis [7,8]. These movements of chloroplasts belong to acclimation mechanisms, which adjust the photosynthetic apparatus to changing light conditions. In darkness, however, there is no uniform distribution pattern of chloroplasts. For example, in the centric diatom *Pleurosira laevis* [9], water angiosperms *Lemna trisulca* [4] and *Halophila stipulacea* [10] as well as terrestrial angiosperms *Tradescantia albiflora* [11] and *Eleusine coracana* [12], chloroplasts are distributed randomly along all walls. By contrast, in prothallial cells (gametophyte) of the fern *Adiantum capillus-veneris* and the moss *Funaria hygrometrica*, chloroplasts are located only along the anticlinal wall [4,13]. Interestingly, in the sporophyte of *A*. *capillus-veneris*, chloroplasts in darkness are distributed evenly along all cell walls [14]. An intermediate model of chloroplast distribution in darkness can be observed in palisade cells of *Arabidopsis thaliana*: along anticlinal walls as well as along the periclinal walls that are internally tangent, while there are no chloroplasts along the periclinal wall neighboring with the epidermis [15,16]. Such a variety of distribution of chloroplasts in darkness makes it difficult to explain the role of chloroplast location in darkness.

If light intensity is low, most of chloroplasts accumulate near the periclinal walls (perpendicular to the direction of light), assuming the so-called face position (accumulation response). In contrast, strong light causes their movement to anticlinal walls (parallel to the direction of light), assuming the so-called profile position (avoidance response) [4,5,17,18,19]. Irradiation with even higher fluence rates can induce atypical clustered distribution of chloroplasts, for example, in aquatic angiosperms *Lemna trisulca and Halophila stipulacea* [20,21], C4 plants *Zea mays* and *Eleusine coracana* [22] as well as succulents *Zygocactus truncatus* and *Kalanchoe fedtschenkoi* [23]. It is presumed that the face position ensures maximization of the amount of energy absorbed by chloroplasts, while the profile position ensures its minimization, thereby protecting the photosynthetic apparatus from excess energy [7,24,25,26,27,28]. The chloroplast clumping under high irradiance, causing self-shading of chloroplasts, is probably also an important photoprotective feature [10].

Special systems detect changes in color, intensity and direction of light in mesophyll cells. In most higher plants, chloroplast movements are induced by blue light and near-UV light [29,30,31,32], absorbed by photoreceptor proteins, phototropins phot1 and phot2 [33,34,35,36]. The accumulation response is controlled by phot1 and phot2, while the avoidance response and dark position, by phot2 [37,38,39]. So far, chloroplast movement has proved to be induced by red light, through the mediation of phytochrome only in cryptogamic plants (including algae *Mougeotia* and *Mesotaenium*, moss *P*. *patens*, ferns *A*. *capillus-veneris* and *Dryopteris filix-mas*) and in one aquatic angiosperm *Vallisneria gigantea* [40,41,42]. In some higher plants, red light participates in chloroplast movements only through the modification of their course [32,43,44,45].

The pathway of light signal transduction has not been identified yet. Potential intracellular messengers are calcium ions [46,47] and enzymes of the phosphatidylinositol pathway [48,49]. In one of the hypothetical models, calcium ions are regarded as a factor controlling the activity of the motor apparatus. Phosphoinositide kinases can play an important role in directional responses of chloroplasts (orientation mechanisms) by controlling the local phosphatidylinositol levels [48,49]. Hydrogen peroxide (H_2_O_2_) may also be an intracellular messenger in directional chloroplast movements, as exogenous H_2_O_2_ was shown to enhance the avoidance response of *Arabidopsis thaliana* chloroplasts [50].

Studies of the mechanism of chloroplast movements controlled by phototropins have shown that the actin cytoskeleton is involved in the motor apparatus [51,52,53,54]. So far, participation of microtubules in chloroplast distribution [55,56,57] has been demonstrated only in mosses (*P*. *patens* and *Funaria hygrometrica*). The mechanism of actin-cytoskeleton-dependent chloroplast movements has not been explained yet. Some authors believe that actin filaments surrounding chloroplasts in the form of a basket and their interactions with cortical actin bundles and myosin are crucial for generation of motive force [17,52,56,58]. According to other researchers, the motive force is generated by blue-light-controlled rearrangements of specific, short actin filaments (the so-called cp-actin) present on the chloroplast surface facing the cell membrane [59,60,61,62,63].

Chloroplast movement may be modified or induced/hampered by factors other than light. So far, researchers demonstrated the impact of physical factors, such as temperature [64,65,66,67,68,69,70], UV radiation [21,71], ultrasounds [72], mechanical stimuli [9,56,73,74], and centrifugation [75], as well as chemical factors: H_2_O and abscisic acid, the water stress hormone [22,23,76,77], NaCl [22], H_2_O_2_ [50], Ca^2+^ and Mg^2+^ ions [46,53,78,79], carbohydrates [80,81], and biotic factors [82]. As in the case of light, chloroplasts may react variously to these factors (avoidance or accumulation responses). Chloroplasts may also form characteristic clusters—like in response to very strong light [21,22,23]. Responses to the same stimulus may vary between plant species. For example, chloroplasts of the fern *A*. *capillus-veneris* protonemata demonstrated mechano-avoidance response, while those of the moss *P*. *patens* showed mechano-accumulation response [56,73,74], while chloroplasts of the centric diatom *Pleurosira laevis* formed clusters around the cell nucleus in response to mechanical stimuli [9]. On the other hand, a variety of factors may cause a similar effect. Light- and mechano-avoidance-like responses were recorded also at low temperature [56,69,70,83] and after H_2_O_2_ treatment [50]. However, the similarity of directional chloroplasts responses to various stimuli does not mean that the mechanism of the responses is uniform. For example, in *A*. *capillus-veneris* external Ca^2+^ [79] was shown to play an important role in the avoidance response induced by a mechanical stimulus, in contrast to the avoidance response triggered by strong light. The study demonstrated that the mechano-relocation movement was sensitive to low concentrations of La^3+^ (plasma membrane Ca^2+^ channel blocker) and Gd^3+^ (a stretch-activated channel blocker).

The above-mentioned studies show that among various environmental factors, metallic elements may also potentially have an impact on directional movement of chloroplasts [79,83]. Among metallic elements we can distinguish trace metals, which are either indispensable for normal functioning of plant cells (Fe, Cu, Mn, Co, Zn, and Ni, although both their excess and deficiency may be harmful) or not used in cellular metabolism (Cd, Pb, Hg, and Al) [84]. Only a few studies of the impact of trace metals on chloroplast movement have been published so far. For example, A1^3+^ ions caused destabilization of the actin cytoskeleton in *Vaucheria* cells, which was connected with the stopping of chloroplast movement [85,86]. Cu^2+^ ions administered exogenously to *Elodea* cells also completely blocked chloroplast movement [83]. Similarly, plastid movement was hampered under the influence of Zn, Pb, and Cd [87] in *Allium* epidermal cells, which contain leucoplasts instead of chloroplasts. Also, the above-mentioned works concerned the impact of trace metals on cells in which plastid movement was not autonomous, but connected with cytoplasmic streaming. There has been no information so far on how the trace elements (in particular the toxic ones) affect light-induced directional chloroplast movement. For cells in which the cytoplasm does not move, the problem was only signaled in experiments with lead-treated protonema cells of the moss *F*. *hygrometrica*, where chloroplasts formed regular clusters in the filamentous cells [88], or a linear pattern distant from cell walls which in this species are one of the most important Pb accumulation sites [89,90]. The cited authors explained the changes in chloroplast distribution with both quantitative and qualitative disturbances in the microtubule cytoskeleton [88,89], since the microtubule cytoskeleton plays a vital role in chloroplast location in protonema cells of mosses [55,57]. However, exposure to light inducing chloroplast movement was not used in their studies, so the relationship between the disturbances and chloroplast movement was not verified.

A question arises whether lead causes changes in chloroplast distribution also in cells of flowering plant. An answer to this question is important because lead is regarded as very dangerous to most living organisms. This results mostly from its high toxicity and widespread distribution in the natural environment [91,92]. Photosynthesis is one of the processes that are the most sensitive to lead treatment. This can be due to disturbed chloroplast morphology and ultrastructure [93,94,95,96,97]. However, so far it has been unclear whether the mechanisms of Pb^2+^ toxic effect on chloroplasts include disturbances in the distribution of these organelles in mesophyll cells.

To study this question, we have selected the small-sized aquatic flowering plant *Lemna trisulca* L., widely used as a model organism for chloroplast movement research [29,46,48,51,80]. In contrast to some other aquatic flowering plants, such as *Vallisneria* or *Elodea*, chloroplast movement in *L*. *trisulca* is not related to cytoplasmic streaming, but it is directional and induced by blue light, like in terrestrial higher plants [29]. The role of the actin cytoskeleton in this process was determined in *L*. *trisulca* with the help of actin-depolymerizing agents, potent inhibitors of blue light-induced chloroplast movements [51]. These movements in *L*. *trisulca* are affected by temperature [64] and ultrasounds [72]. They are controlled by calcium ions [46,47], phosphoinositides [48] and carbohydrates [80].

On the basis of changes in chloroplast distribution in cells of moss *F*. *hygrometrica* resulting from treatment with lead [88,89], we hypothesized that this metal causes changes in chloroplast distribution also in flowering plants. To verify this hypothesis, we decided to determine whether the toxic effect of lead on the photosynthetic apparatus of the aquatic angiosperm *Lemna trisulca* includes disturbances in directional responses of chloroplasts and chloroplast distribution patterns. In order to do so, we (1) analyzed quantitatively and qualitatively chloroplast distribution in mesophyll cells of control and lead-treated plants in darkness; (2) compared the final effects of chloroplast movements in darkness and in weak or strong white light; (3) checked whether lead affects blue light-induced chloroplast responses (their amplitudes and rates); (4) checked whether the spatial distribution of chloroplasts in mesophyll cells is related to lead location; (5) checked whether lead-induced changes in chloroplast location are typical of avoidance or accumulation strategy. The contribution of additional factors, which might help to explain the mechanism(s) of the effect of lead on chloroplasts, was also analyzed: (a) levels of H_2_O_2_ in *L*. *trisulca* cells, and (b) changes in actin cytoskeleton structure. This stemmed from the finding that H_2_O_2_ participates in the induction of changes in chloroplast location [50] and an increase in H_2_O_2_ concentration may be caused by lead [98]. In view of the above, the role of catalase (H_2_O_2_-specific scavenger) was also taken into account when analyzing chloroplast response to lead. Changes in chloroplast distribution may also result from changes of the actin cytoskeleton structure [59,60,61]. For lead ions, no published information on the impact of the metal on the actin cytoskeleton was available.

## Materials and Methods

### 1.1. Plant material

**Fig 1:**
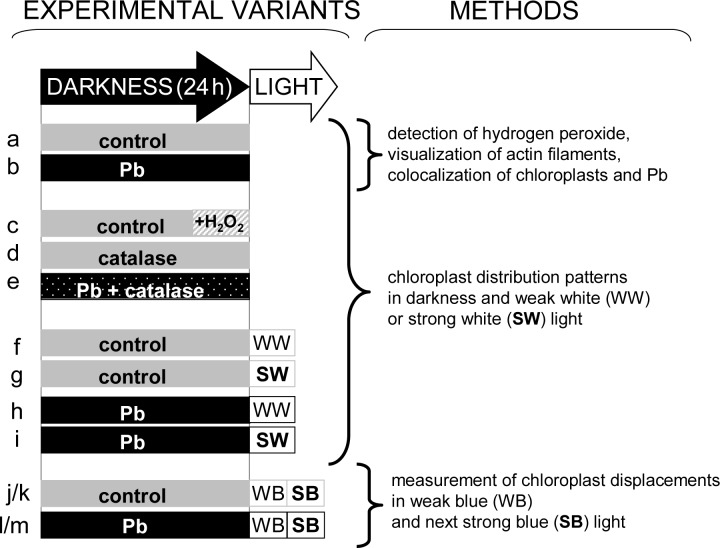
Experimental design and methods used in individual experimental variants. *Lemna trisulca* plants incubated in darkness for 24 h in control medium (a), with 15 μM Pb^2+^ (b), with 10^–3^M H_2_O_2_ added for the last 3 h of incubation (c), with 100 U catalase (d), or with Pb and catalase simultaneously (e); plants pre-incubated in darkness for 24 h and then exposed to white light for 1 h (weak 50 μE m^-2^s^-1^ or strong 1000 μEm^-2^s^-1^) in control medium (f-g) or with Pb (h-i); plants pre-incubated in darkness for 24 h and then exposed to blue light (for 45 min weak 2 μE m^-2^s^-1^ and next for 45 min strong 113 μE m^-2^s^-1^) in control medium (j-k) or with Pb (l-m).

The culture of the water plant *Lemna trisulca* L. (star duckweed) originated from the collection of the Jagiellonian University Botanical Garden in Kraków, Poland. *Lemna* was cultured in a greenhouse in Erlenmeyer flasks on a liquid medium according to Wang [99], at pH 5.7, with a short-day photoperiod (10 h L : 14 h D) at 23±1°C and a photosynthetic photon flux density of 70 μmol m^-2^ s^-1^. Plants were moved to a fresh medium every 3 weeks. Experimental design is presented in Fig. 1.

### 1.2. Experimental variants in darkness

In contrast to the most widely recognized *Lemna minor* L. (common duckweed), *L*. *trisulca* specimens grow under the surface of water [100]. Since the plant is entirely submerged, trace metal ions in the medium are available throughout the plant surface. Therefore, in contrast to most land plants, Pb ions may come in direct contact with photosynthetic tissue. All experiments were performed on young *L*. *trisulca* fronds.

For the experiments, morphologically similar individuals were transferred to 30 mL of 50-fold diluted Wang medium with lead nitrate [15 μM Pb^2+^ as Pb(NO_3_)_2_] for 24 h in darkness (Fig. 1). Control plants were also kept in darkness for the same period in 30 mL of 50-fold diluted Wang medium. Some experiments were carried out in the same conditions for 24 h with 100 U of catalase in the medium with or without lead (Fig. 1). In the experiments analyzing the impact of H_2_O_2_ on chloroplast distribution, the plants were incubated in darkness for 21 h in the control medium, and subsequently for 3 h in the medium with 10^–3^ M H_2_O_2_. The plants from all the above variants were then collected for microscopic analysis of chloroplast distribution (Fig. 1). Some of the plants treated with lead in darkness (as well as those from analogous control conditions) were used to analyze H_2_O_2_ in fronds (see section *1*.*7*. and *1*.*8*.), lead and chloroplast colocalization (see section *1*.*9*.), actin cytoskeleton structure (see section *1*.*6*.), and further stages of experiments with light (see section *1*.*3*., Fig. 1).

### 1.3. Experimental variants in light on dark pre-adapted plants

Some control plants and plants treated with lead for 24 h in darkness were subsequently exposed to white or blue light (Fig. 1). Plants treated with white light were intended for microscopic analyses of chloroplast distribution (Fig. 1). Blue light was used for recording chloroplast movements (see section *1*.*5*.), because blue wavelengths are responsible for inducing the movement of chloroplasts in *L*. *trisulca*. In view of the specific measuring technique used, it was also necessary to distinguish wavelengths inducing chloroplast movement (blue light) and the measuring light (red).

Two intensity variants were used for white light. Plants incubated in darkness in the presence/absence of lead were exposed to 50 or 1000 μE m^-2^s^-1^ white light produced by a 100 W, 12 V halogen lamp for 1 h (Fig. 1). The distribution of chloroplasts in mesophyll cells was recorded immediately after exposing the plants to light in a confocal microscope (section *1*.*4*.).

Young fronds, dark-adapted for 24 h (control or Pb-treated), were exposed to continuous weak (2 μE m^-2^s^-1^) for 45 min and then to continuous strong (113 μE m^-2^s^-1^) blue light for next 45 min (Fig. 1). The plants were exposed to light in a photometer, and the respective accumulation and avoidance responses were measured as leaf transmittance changes (see section *1*.*5*.).

### 1.4. General chloroplast distribution

To analyze chloroplast distribution in *L*. *trisulca* mesophyll cells, plants from experimental variants (treated with lead alone, with lead and catalase, or with catalase alone for 24 h in darkness, or with H_2_O_2_ for the last 3 h of incubation in darkness) were compared with control material. Additionally, chloroplast responses in darkness were compared to responses to weak and strong white light (section *1*.*3*.) to check whether light is a factor modifying chloroplast response to lead.

*L*. *trisulca* fronds were placed on microscope slides. We analyzed mesophyll in the apical areas of fronds composed of a single layer of cells. The 3-dimensional positions of chloroplasts were imaged by their autofluorescence with a confocal microscope LSM 510 (Zeiss, Jena, Germany) attached to an Axiovert 200M (Zeiss, Jena, Germany) inverted microscope using a LD Plan-Neofluar 63× lens. Chloroplast autofluorescence was excited using a 633-nm laser (25% 5 mW, He-Ne ions) with emission above 650 nm. Images were collected over an area with pixel spacing of 0.2 μm. Each optical section was averaged over 20–30 frames and sampling was repeated with a focus motor increment of 1.1–1.5 μm through the whole frond.

Projection and DepthCod features of LSM510 software (Zeiss, Jena, Germany) were used for 3D image reconstruction of chloroplast distribution in cells, while Orthogonal section display mode was used for the imaging of chloroplast location in cross-sections of mesophyll cells (S1 Fig.). The share of chloroplasts along anticlinal and periclinal walls was calculated both for profile and face positions on the basis of microscopic images. This was done to determine to what extent chloroplast responses to Pb^2+^ resemble avoidance (profile position), accumulation (face position) or orientation in darkness [29].

Calculations were made on the basis of 3 replicates of 3 plants each and at least 3–4 cells of each plant (about 30 cells in total).

### 1.5. Measurement of chloroplast movement *in vivo* (photometric method)

To check the impact of lead on the dynamics of changes taking place during chloroplast movement, plants pre-incubated in darkness were then exposed to constant weak blue light (2 μE m^-2^ s^-1^) for 45 min, and subsequently to strong blue light (113 μE m^-2^ s^-1^) for next 45 min.

A double-beam photometer [11] was used for quantitative measurement of chloroplast displacements. The method is based on changes in transmission of monochromatic red light (λ = 660 nm, 0.1 μmol m^-2^ s^-1^). Incident light is perpendicular to the leaf surface. Fluence rates were measured with a silicon photodiode calibrated against a LI-COR quantum meter (LI-COR, Lincoln, NB, USA). The actinic light was obtained from a 100 W, 12 V halogen lamp with the following combinations of filters: for blue light BG12, BG23, GG13; for red light RG1, a heat absorbing filter C805 and a dichroic short-pass filter 710 nm (Schott, Jena, Germany). Neutral density filters were used to reduce fluence rates.

Changes in light transmission accompanying chloroplast responses were recorded, and their characteristic amplitudes and velocities were analyzed according to Banaś and Gabryś [80]. Calculations were made on the basis of 3 replicates of 10 plants each.

### 1.6. Fluorescent labeling of the actin cytoskeleton

Actin filaments were visualized by the modified glycerol permeation method of Olyslaegers and Verbelen [101]. Apical parts of *L*. *trisulca* fronds were cut into pieces of about 2 mm × 3 mm. Immediately after cutting, the frond fragments were incubated in a staining solution containing actin-stabilization buffer [50 mM piperazine-N,N′-bis(2-ethanesulfonic acid) (PIPES), 10 mM ethylene glycol tetraacetic acid (EGTA), 5 mM MgSO_4_, pH 6.9] with 3% (w/v) glycerol and 0.02 μM Alexa Fluor 488 phalloidin (Invitrogen/Molecular Probes). The samples were incubated in the staining solution in darkness for 3 h at 15°C. Frond fragments were then placed on microscope slides (in the actin-stabilization buffer containing 3% glycerol) and observed immediately under the microscope. Fluorescence images of microfilament organization in the mesophyll were observed on the basis of a series of optical sections and captured with a LSM 510 confocal microscope (Zeiss, Jena, Germany). The samples were excited at 488 nm using a krypton-argon laser line and detected using a 505–530 nm bandpass filter. To quantify the differences in microfilament morphology, first the maximum intensity projections of stacks of confocal images were obtained. The images were then transformed by thresholding segmentation and converted into binary images. Individual actin filament bundles and branches surrounding chloroplasts were marked and then we determined the following parameters: fiber length, fiber width (to characterize the size of microfilaments) and form factor (to characterize their shape). Fiber length and fiber width are defined as:

$$\mathrm{Fiber length}=\frac{\mathrm{PerimeterF}+\sqrt{\mathrm{Perimeter}F^{2}-16\mathrm{AreaF}}}{4}$$

$$\mathrm{Fiber width}=\frac{\mathrm{PerimeterF}-\sqrt{\mathrm{Perimeter}F^{2}-16\mathrm{AreaF}}}{4}$$

where *PerimeterF* and *AreaF* are the perimeter and area of the filled region, respectively. These parameters were measured in pixels. Form factor provides information about the shape of objects. It is calculated by software according to the following formula: 4π·area/perimeter^2^. When the form factor is close to one, the objects are close to spherical, and the factor becomes lower with the shape elongation [52]. With the use of software package KS300 3.0 (Carl Zeiss Vision, Germany), the calculations were made on the basis of 3 replicates of 3 plants each and at least 3–5 cells of each plant (about 35 cells in total).

### 1.7. Detection of hydrogen peroxide release in plants treated with lead

Accumulation of H_2_O_2_ in *L*. *trisulca* was detected by staining with dichlorodihydrofluorescein diacetate (DCFH-DA, Sigma-Aldrich), as described by Małecka et al. [102]. Fresh plants were submerged in 4 μM DCFH-DA dissolved in dimethylsulfoxide (DMSO) in 50 mM potassium phosphate buffer, pH 7.4, for 2 h. The fronds were washed twice with the loading buffer and then observed under a fluorescence stereomicroscope SteREO Lumar V12 (Zeiss, Jena, Germany) with a filter set Lumar 38 HE (excitation 470/40 nm, emission 525/50 nm) and objective NeoLumar 0.8×. Images were analyzed with AxioVision software, version 4.2 (Zeiss, Jena, Germany). Microscopic analyses were based on 3 replications of 10 plants each.

### 1.8. Determination of hydrogen peroxide concentration in plants treated with lead

To confirm that the change in fluorescence density of reaction with DCFH-DA are related to changes in H_2_O_2_ concentration, quantitative analysis was also performed. Its concentration in control plants and in plants treated for 24 h with Pb in darkness was determined spectrophotometrically, as described by Becana et al. [103]. Plant samples were prepared in compliance with the procedure described by Mai et al. [104]. The decrease in absorbance was measured at a wavelength of 508 nm (*A*_508_) in the Perkin Elmer Lambda 15 UV-Vis spectrophotometer (Norwalk, CT). H_2_O_2_ content was determined from the difference in *A*_508_ between each sample and blank probes containing 5% trichloroacetic acid. In order to make the standard curve, H_2_O_2_ (30%, Sigma-Aldrich) was diluted to 0.5–20.0 μM. Its concentration was expressed as μmol H_2_O_2_ g^-1^ fresh weight (FW). Measurements were based on 3 replications of 0.5 g of fresh matter each (about 90 plants).

### 1.9. Colocalization of lead, calcium, and chloroplasts with the help of X-ray microanalysis

Lead taken up by plants was localized by using a transmission electron microscope JEM 1400 (JEOL Co., Tokyo, Japan) supplied with energy-dispersive X-ray microanalysis system (Oxford Instruments) and high resolution digital camera (CCD MORADA, SiS-Olympus) for X-ray mapping microanalysis of chemical elements (for more details, see [105]).

This method was used for colocalization of lead and chloroplasts. We compared lead content in areas neighboring chloroplasts (cell wall + cell membrane + a thin layer of cytoplasm between chloroplasts and cell membrane) and in areas with no chloroplasts. Frame analyses of 40 μm^2^ size were conducted at an accelerating voltage of 80 keV. The time of registration of signals forming the spectrum was 120 min for the frame analysis. Lead concentration was assessed on the basis of the peak area for the emission line Lα (10540 keV). Additionally, calcium content was determined in these places on the basis of the Kα emission line (4050 keV). Element content data were processed using Link software for assessment of apparent atomic concentration (at%). Calculations were made on the basis of 3 replications of 10 cells from 3 plants each.

### 1.10. Statistical analysis

The analysis of variance (ANOVA) was applied to verify whether means from independent experiments within a given experimental variant were significantly different at *p* < 0.05. Statistical significance of pair-wise differences between mean values was analyzed as elementary contrasts. The statistical analysis was performed using statistic package Genstat 15.

## Results

### 2.1. Distribution of chloroplasts in darkness

**Fig 2:**
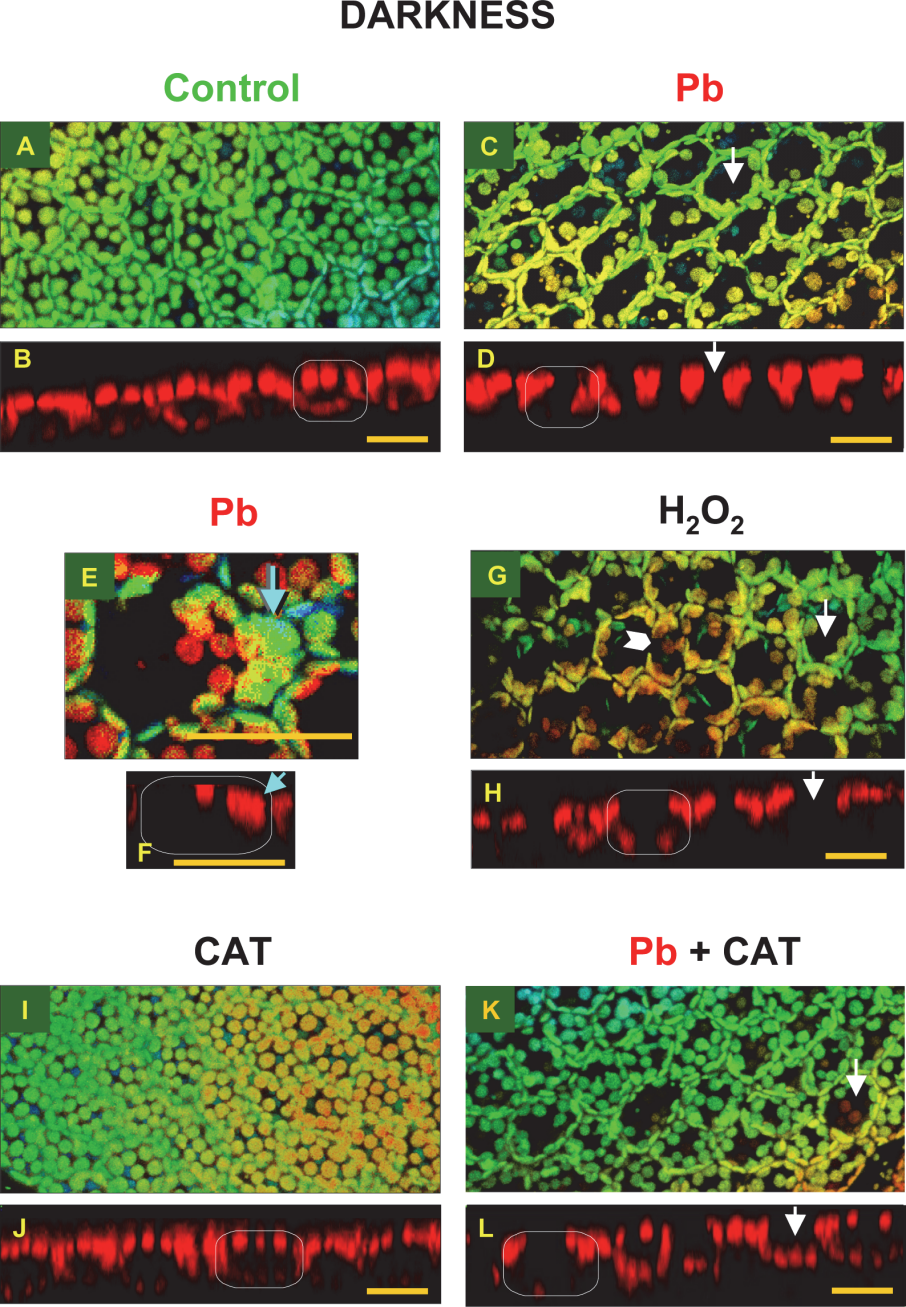
Distribution of chloroplasts in mesophyll cells of *Lemna trisulca* fronds in darkness. In control plants (A, B) and in plants treated with lead (15 μM Pb^2+^; C-F), hydrogen peroxide (10^–3^ M H_2_O_2_; G, H), catalase (100 U; I, J) or simultaneously with lead and catalase (15 μM; K, L) for 24 h (in the case of H_2_O_2_, plants were treated with it for the last 3 h only). The larger micrographs show 3D reconstructions of frond fragments, obtained on the basis of a series of optical sections adjacent to the surface of fronds in a confocal microscope (S1 Fig.). The reconstructions include chloroplasts from a single layer of mesophyll cells (in some places tiny epidermal chloroplasts are also visible). A color scale is used to highlight the image depth (warm colors represent chloroplasts located closer to the viewer, while the cool ones, the more distant organelles). The smaller micrographs show chloroplast distribution (red autofluorescence) in cell cross-sections in single optical sections (S1 Fig.). The white rectangle presents a schematic outline of a cross-section of a single cell. The white arrows indicate chloroplast-free areas visible in 3D reconstruction and the corresponding locations in cross-sections. Blue arrows (E, F) indicate places in which chloroplast clusters are present, while the white arrowhead (G) points to the area neighboring the anticlinal wall, where chloroplasts were absent. Scale bar = 20 μm.

**Fig 3:**
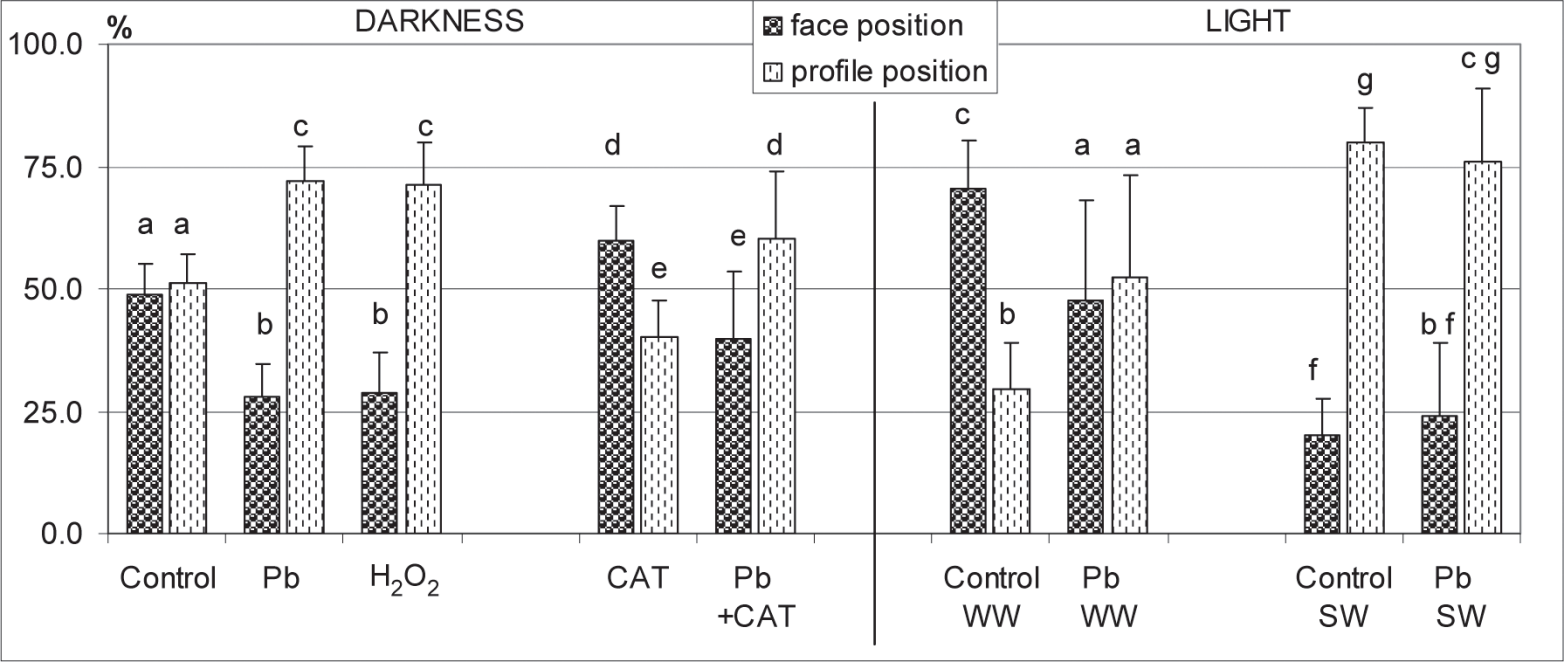
Percentage of chloroplasts adopting the face or profile position per mesophyll cell of *Lemna trisulca* in various experimental variants. During incubation, control plants and plants treated with lead (15 μM Pb^2+^) were placed in darkness for 24 h, weak white light (WW; 50 μE m^-2^s^-1^, 1 h after 24 h in darkness or strong white light (SW; 1000 μEm^-2^s^-1^, 1 h after 24 h in darkness). In two variants, plants were treated with catalase (100 U, CAT) alone or with Pb in darkness for 24 h. For comparison with the reaction to lead, some plants were treated with H_2_O_2_ alone (10^–3^ M) for the last 3 h in darkness. Different small letters (a-g) denote statistically significant differences (*p* < 0.05).

**Fig 4:**
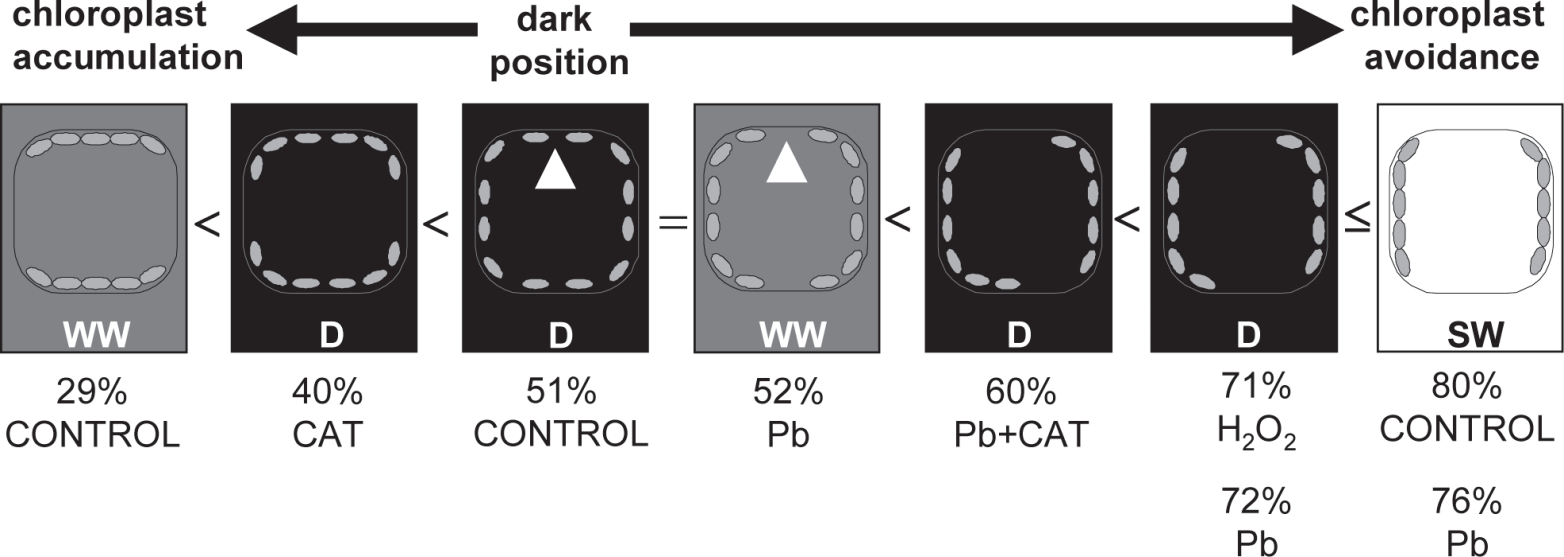
Summary of results shown in Figs. 2–5. A diagram of chloroplast distribution in cross-sections of *Lemna trisulca* cells of control plants and plants treated with lead, hydrogen peroxide (H_2_O_2_), catalase (CAT), or simultaneously with lead and catalase in darkness (D) and weak or strong white light (WW or SW, respectively). The numbers show the percentage of chloroplasts in the profile position in the particular variants (in ascending order). Two numbers under a cell indicate an absence of significant differences between two experimental variants. The symbol ≤ denotes that differences between Pb-SW and both Pb-D and H_2_O_2_-D are not significant but they are significant between control-SW and both Pb-D and H_2_O_2_-D. Also control-D and Pb-WW did not differ in the number of chloroplasts in the profile position, but the variants were illustrated separately in view of a difference in chloroplast distribution (marked with white arrowheads). The diagram shows three basic chloroplast positions in control variants: chloroplast accumulation (in WW light), dark position (in D), and chloroplast avoidance (in SW).

**2.1.2. Control plants**. In control plant cells, growing for 24 h in darkness in the control culture medium, chloroplasts were distributed in a single layer, more or less uniformly along all the cell walls (Figs. 2 A and B, 3 and 4). Most chloroplasts did not touch each other, and relatively large free areas were seen between them. Plastids located along periclinal walls (parallel to frond surface) were seen in the so-called face position (i.e. top view, with their largest surface visible), while those located along anticlinal walls (perpendicular to frond surface) were observed in the so-called profile position (i.e. side view). The percentage of organelles in each position was similar, 48.9% and 51.1%, respectively (Fig. 3). The uniform distribution of chloroplasts along all the walls reflected the pattern of the walls in the cell and thus marked the cell shape (walls were not visible in the chloroplast detection method used).

**2.1.3. Lead**. The micrographs of fronds treated with lead (15 μM Pb^2+^, 24 h in darkness) showed considerable differences in the distribution of chloroplasts, as compared to control plants (Figs. 2 C and D, 3 and 4). As many as 72.2% of them were seen along anticlinal walls in the profile position (Fig. 3). The remaining chloroplasts in the face position (27.8%) were unevenly distributed along periclinal walls. Most of them were located at the edges of the walls, near their contact point with the anticlinal walls, and only a few were seen along the central part of the periclinal walls. Thus the lumen of the cell, when viewed from the top, made an impression that most chloroplasts moved to the sides of the cell (Figs. 2 C and D, and 4). In the majority of cases, chloroplasts formed a single layer, in which they adhered to each other. However, sometimes the organelles overlapped, locally creating two layers or irregular clusters (Fig. 2 E and F).

**2.1.4. Hydrogen peroxide**. After treating plants with 10^–3^M H_2_O_2_ in darkness for the last 3 h of the 24-h incubation, the chloroplasts adopted a different position than those in the control plants. Most of them (71.4%) were seen in the profile position like after lead treatment (72.2%; Figs. 2 G and H, 3 and 4). However, in comparison with the plants treated with lead, their distribution along the walls was slightly different—the chloroplast pattern along anticlinal walls was less regular. For example, chloroplasts were absent in some parts of walls (Fig. 2G), while clusters of the organelles were seen in other areas. The percentage of chloroplasts in the face position (28.6%) was nearly as low as for the plants treated with lead (Fig. 3). Chloroplasts were located along the central part of periclinal walls as rarely as in the plants treated with lead.

**2.1.5. Catalase**. The application of catalase (100 U, 24 h in darkness) caused a change in the distribution of chloroplasts in comparison to their position in all the experimental variants presented earlier (control, Pb, H_2_O_2_; sections *2*.*1*.*2*.-*4*.). In the presence of catalase, the share of chloroplasts in the face position (59.7%) was significantly higher than the percentage of chloroplasts in the profile position (40.3%; Fig. 3) and about 1.2-fold higher than in control plants, while as much as 2.1-fold higher than in the cells treated with lead. Numerous chloroplasts in the face position uniformly filled the spaces along periclinal walls, like in control plant cells (Fig. 2 I and J, compared with Figs. 2 A and B, and 4). No chloroplast-free areas were present in spaces neighboring the central area of the periclinal walls, similar to control cells (opposite to responses to Pb and H_2_O_2_). A small difference in relation to control plants was only that the chloroplasts were more densely spaced along periclinal walls. The situation along the anticlinal walls was opposite: the accumulation of chloroplasts along these walls was smaller in the plants treated with catalase than in the control plants.

**2.1.6. Lead and catalase**. Interesting results were obtained for plants treated simultaneously with lead (15 μM) and catalase (100 U) for 24 h in darkness. The percentage of chloroplasts in the face and profile positions was opposite than in the plants treated with catalase alone. Chloroplasts in the profile position dominated (60.3%, compared to 40.3% in the variant with catalase alone; Fig. 3), while the share of chloroplasts in the face position was significantly smaller (39.7%, compared to 59.7% in the variant with catalase alone). In the presence of catalase and Pb, the share of chloroplasts in the profile position was about 1.2-fold higher than in control plants, and obviously about 1.2-fold fewer chloroplasts occupied the face position than in control plants (Fig. 3). In microscopic images of plants treated simultaneously with lead and catalase, dispersion of chloroplasts in the face position along periclinal walls was recorded in most cases (Figs. 2 K and L, and 4). Most often, the dispersion had the form of local areas in which chloroplasts were partially scattered into the sides of the cells. Very rarely they moved completely towards anticlinal walls (opposite to the cells treated with lead). Free spaces between chloroplasts were usually seen along one of the periclinal walls of a given cell—outer or inner—and less often they covered both walls. This was yet another difference to the chloroplast pattern in plants treated with lead (Fig. 2 K and L, compared with Fig. 2 A and B).

### 2.2. Distribution of chloroplasts in white light

The presented distribution of chloroplasts in plants treated with lead in darkness resembled the avoidance response described in literature (i.e. the avoidance of strong light by chloroplasts), so additional experiments involving light were performed to find out if the response to lead was similar to the response to light. Both strong white light and an intermediate variant with weak white light were used.

**Fig 5:**
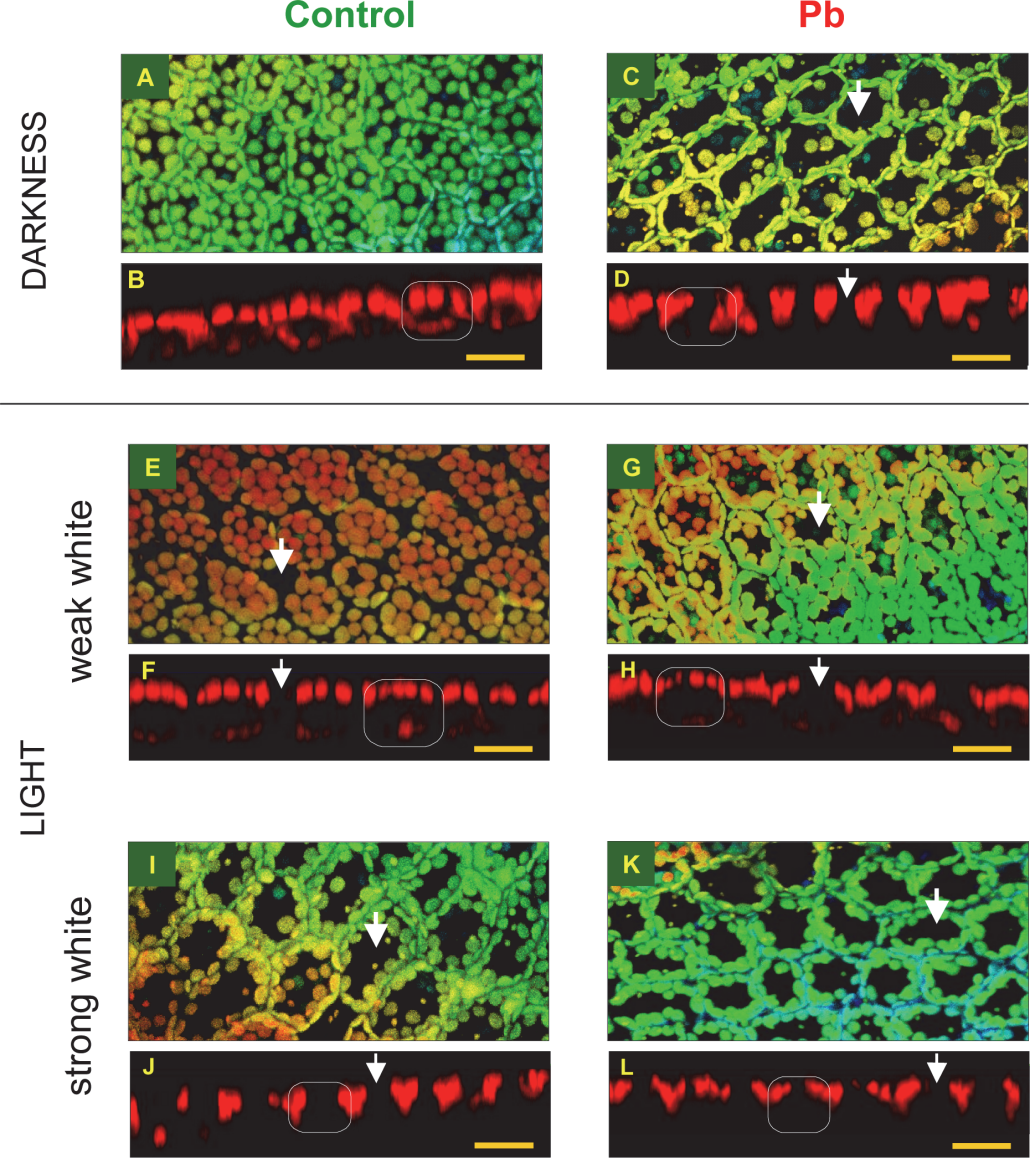
Distribution of chloroplasts in mesophyll cells of *Lemna trisulca* in white light. Control plants (A, B, E, F, I, J) and plants treated with lead (15 μM Pb^2+^; C, D, G, H, K, L) for 24 h in darkness (A-D), followed 1 h by weak white light (E-H; 50 μE m^-2^s^-1^) or strong white light (I-L; 1000 μEm^-2^s^-1^). Other explanations as in Fig. 2 and S1 Fig. Scale bar = 20 μm.

**2.2.1. Weak white light and control medium**. In cells of control plants that grew for 24 h in darkness and for the next 1 h in weak white light (50 μE m^-2^ s^-1^), most chloroplasts (about 70.6%) were in the face position (Figs. 3, 5 E and F, and 4), and only 29.4% were in the profile position—a situation exactly opposite to that recorded for the plants treated with Pb and H_2_O_2_ in darkness (Fig. 3). Chloroplasts in the face position were distributed uniformly in a single layer, similar to dark position. In contrast, the space between them was much lesser than in darkness, and they formed more dense patterns (Fig. 5E). In comparison with the dark position (Fig. 5 A and B), the significant increase in the number and density of chloroplasts along periclinal walls seen in weak light in control plants was an element of chloroplast accumulation response. In contrast to the control plants treated with strong light (Fig. 5 I and J, section *2*.*2*.*3*.) or with Pb (Fig. 5 C and D) and H_2_O_2_ in darkness (Fig. 2 G and H, sections *2*.*1*.*3*.-*4*.), the dispersion of chloroplasts took place along anticlinal and not periclinal walls. In the central areas of anticlinal walls, chloroplasts were absent or seen sporadically only (Fig. 5F). In 3D microscopic reconstructions, areas without chloroplasts were often seen in peripheral cell parts along anticlinal walls (Figs. 5 E and F, and 4), especially at contact points of several mesophyll cells (Fig. 5 E and F—white arrow). Therefore, the chloroplast pattern did not mark cell boundaries as was the case in darkness (Fig. 5E compared with Fig. 5A).

**2.2.2. Weak white light and lead**. The distribution of chloroplasts in plants treated with lead (15 μM Pb^2+^) for 24 h in darkness and next 1h in weak white light (50 μE m^-2^s^-1^) was very different from that recorded for control plants. The percentage of chloroplasts in the face and profile positions was almost the same: 47.5% and 52.5%, respectively (Fig. 3). The number of chloroplasts in the face position was thus about 1/3 lower from that in control plants in weak light. Interestingly, the face/profile levels were almost identical to those for control, dark-adapted plants (Fig. 3). However, qualitative differences in chloroplast distribution were discovered between both groups. In the presence of lead in weak light, chloroplasts along periclinal walls were not distributed evenly, as was the case in control, dark-adapted plants (Fig. 5 G and H compared with Figs. 5A, B, and 4). Most of them formed dense patterns in the face position only at the edges of periclinal walls adjacent to anticlinal walls. Chloroplasts were dispersed or absent from the central parts of the walls, in contrast to the pattern observed in control plants (both exposed to weak and/or strong light). Chloroplasts were usually absent only from one of the periclinal walls, and, more rarely, from both of them (Fig. 5 G and H). The allocation of chloroplasts along these walls was therefore not as full as in a typical avoidance response (e.g. Figs. 5 I and J, and 4) for two reasons. Firstly, chloroplasts avoided only one of the periclinal walls, and, secondly, only the central part of the wall. The pattern of chloroplasts along anticlinal walls was uniform. The distribution of chloroplasts along the walls was therefore more similar to that recorded for control plants in darkness than to that seen in control plants in weak light (Fig. 5 G and H compared with Figs. 5 A, B, E, F, 3 and 4).

**2.2.3. Strong white light and control medium**. In control plants moved from darkness to strong white light (1000 μE m^-2^s^-1^), after 1 h a significant change in chloroplast location, characteristic for avoidance response, was recorded. Most chloroplasts, i.e. about 79.7%, were in the profile position (compared to 51.1% in darkness; Fig. 3), only 20.3% stayed in the face position (compared to 48.9% in darkness). The chloroplasts formed uniform, dense patterns along anticlinal walls, while their distribution was uneven along periclinal walls (Figs. 5 I and J, and 4). Chloroplasts in the face position were distributed mainly at the edges of periclinal walls, near their contact with anticlinal walls. They were most often absent from the central part of the walls, and only sporadically individual chloroplasts were seen there (Fig. 5 I and J). The size of the chloroplast-free areas was similar to that recorded for plants treated with lead in darkness (compared with Fig. 5 C and D). However, they were bigger than those in the plants treated with lead in weak light (Fig. 5 I and J compared with Figs. 5 G, H, and 4).

**2.2.4. Strong white light and lead**. In the cells of plants treated with lead for 24 h in darkness and then exposed to strong white light for 1 h, distribution of chloroplasts was very similar to that in strong light-exposed control plants. Most chloroplasts (about 76.1%), were seen in profile, while only about 23.9% in the face position (Fig. 3). The manner of distribution of chloroplasts along the walls was very similar to that recorded for control plants in strong light. Most chloroplasts formed dense patterns along anticlinal walls. Only a few of them remained along periclinal walls—mainly at their edges, neighboring with anticlinal walls (Fig. 5 K and L, and 4). Thus, it was a characteristic avoidance response pattern, as that seen in the corresponding control plants. Similar images were also seen in darkness, when the plants were additionally treated with Pb or H_2_O_2_ (compared with Figs. 5 C, D and 2 G, H; Fig. 3).

### 2.3. Directional responses of chloroplasts in blue light

**Fig 6:**
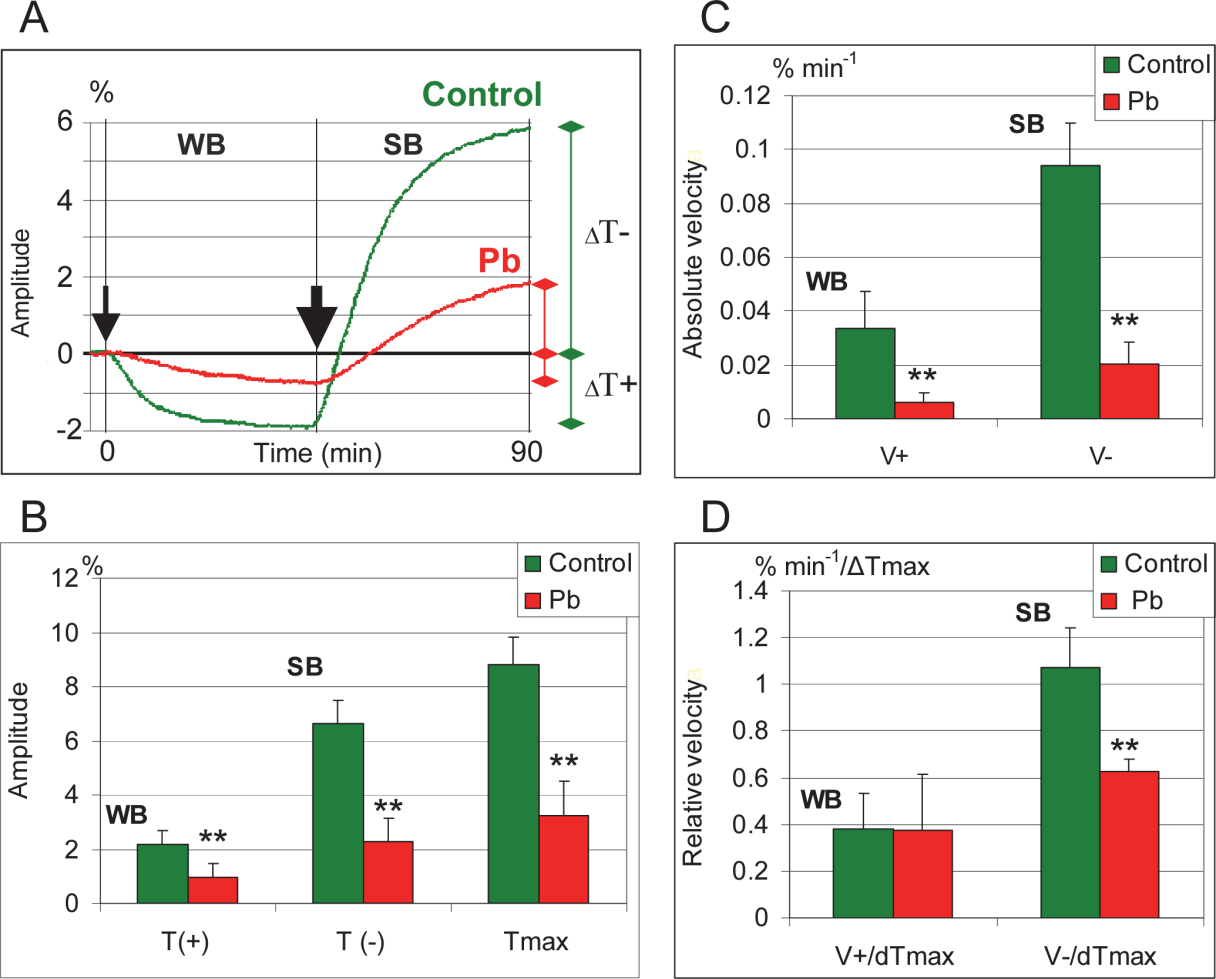
Chloroplast displacements in mesophyll cells of *Lemna trisulca* in blue light. Weak blue (WB, 2 μE m^-2^s^-1^) and strong blue light (SB, 113 μE m^-2^s^-1^) in the presence of lead (15 μM Pb^2+^) and in control plants. The line graph (A) illustrates changes in the location of chloroplasts (recorded on the basis of red light transmittance) during 45 min of exposure to constant weak blue light (a thin arrow marks when the light was turned on; darkness was the starting position) and during 45 min of exposure to strong blue light (a thick arrow marks when the strong light was turned on). The column diagrams (B-D) show amplitude of accumulation [ΔT(+), marked as T(+)] and avoidance [ΔT(-), marked as T(-)] as well as the maximum amplitude [ΔT_max_ = ΔT(-) + ΔT(+), marked as Tmax]; absolute velocity (% min^-1^) of accumulation [V(+)] and avoidance [V(-)]; and relative velocity (% min^-1^/ΔTmax) of accumulation [V(+)] and avoidance [V(-)]. **p < 0.05.

**2.3.1. Weak blue light and control medium**. Fronds of control plants previously adapted to darkness showed a decrease in the measured light transmittance under the influence of weak blue light (2 μE m^-2^s^-1^) (Fig. 6A). This was due to an increased accumulation of chloroplasts along periclinal walls. The decrease in light transmittance was fastest during the first 15 min of plant exposure to weak blue light and gradually decreased thereafter. After 45 min, the light transmittance level expressed by the value of amplitude ΔT(+) was about 2% lower than that recorded for the plants growing in darkness (Fig. 6B).

**2.3.2. Strong blue light and control medium**. Following exposure to weak blue light, the control plants were subsequently exposed to strong blue light for 45 min. The change in blue light intensity caused an avoidance response of chloroplasts, manifested in an increased light transmittance (Fig. 6A). A particularly fast increase in light transmittance was recorded during the first 15 min of exposure, as reflected in the steep inclination of the curve at the stretch corresponding to that period. Within 45 min, the light transmittance expressed by the amplitude ΔT(-) increased by about 6.7% in relation to the dark level (Fig. 6B). The maximum amplitude of light transmittance between the extreme chloroplast positions, constituting the total of ΔT(-) and ΔT(+), reached almost 8.8%. The rate of the chloroplast avoidance response caused by the strong blue light, expressed both in absolute values and in values normalized in relation to the maximum amplitude, was 3-fold higher than that recorded for the accumulation response in weak blue light (Fig. 6C and D).

**2.3.3. Weak blue light and lead**. Exposure of the plants treated with lead to weak blue light caused chloroplast accumulation response, like in control plants (Fig. 6A). The amplitude and rate of the response, expressed in absolute values, were significantly lower (2-fold and 4.5-fold, respectively) than those recorded for control plants (Fig. 6B and C). The difference in the rate of chloroplast accumulation response was not statistically significant when calculated in relative values, normalized to the maximum amplitude (Fig. 6D). The transmittance curve did not show any clear stage of fast decrease during the first 15 min in control plants. Changes in transmittance were almost uniform during the entire period of exposure to weak blue light, as expressed in the flattening of the curve.

**2.3.4. Strong blue light and lead**. In response to strong blue light in the presence of lead, the values of amplitude ΔT(-) were significantly smaller (3-fold) than in the control plants (Fig. 6B). The rate of chloroplast avoidance response, both in absolute values and normalized to ΔT_max_, was also significantly smaller (4.5-fold and 1.7-fold, respectively) than in control plants (Fig. 6C and D). Differences with the control plants also included the shape of transmittance curves. In the presence of lead there was no rapid increase in light transmittance during the first 15 min typical of the control plants (Fig. 6A). The first stage of faster increase in transmittance was almost twice as long (about 30 min) as in control plants. During that time, the transmittance changed more slowly than in control plants, as evidenced by smaller curve inclination angles. It should be noted that light transmittance curves were flatter than those recorded for control plants throughout the experiment (Fig. 6A).

### 2.4. Actin microfilaments (in darkness)

**Fig 7:**
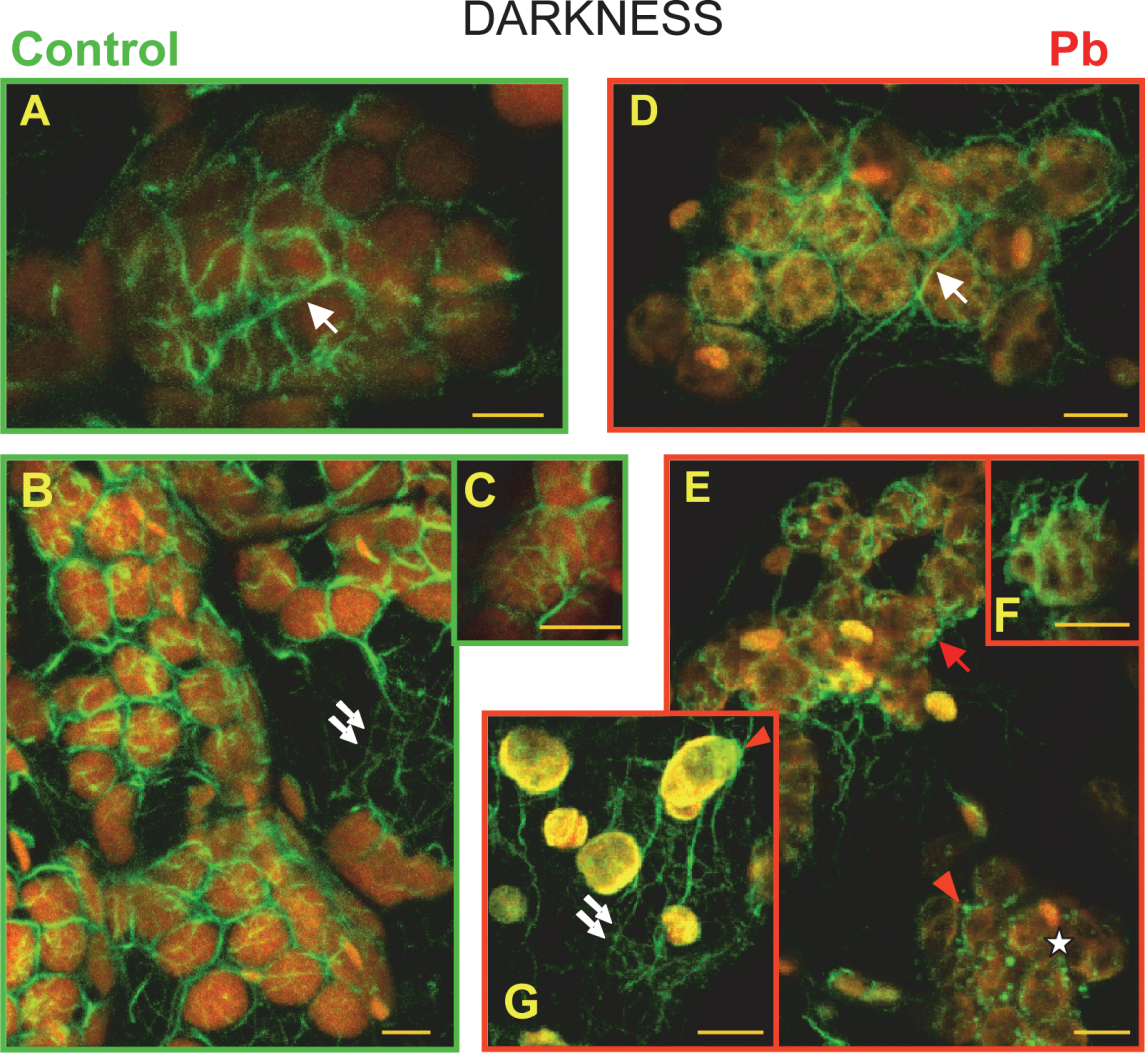
Visualization of actin filaments *Lemna trisulca* mesophyll cells. With the help of Alexa Fluor 488—phalloidin (green fluorescence) in control plants (A-C) and plants treated with lead (D-G) in darkness. Chloroplasts are visible thanks to the red autofluorescence of chlorophyll. The network of microfilament bundles varying in thickness, which were not directly connected with chloroplasts, is marked with double arrows. Microfilament bundles twisting around plastids (single white arrows), with their branches, are also marked (C and F). In cells treated with lead, disturbances in microfilament pattern were recorded: absence of long bundles twisting around plastids (asterisk), fragmentation of bundles twisting around chloroplasts (red arrow), and thick, local accumulations of actin (red arrowhead). Scale bar = 5 μm

**Fig 8:**
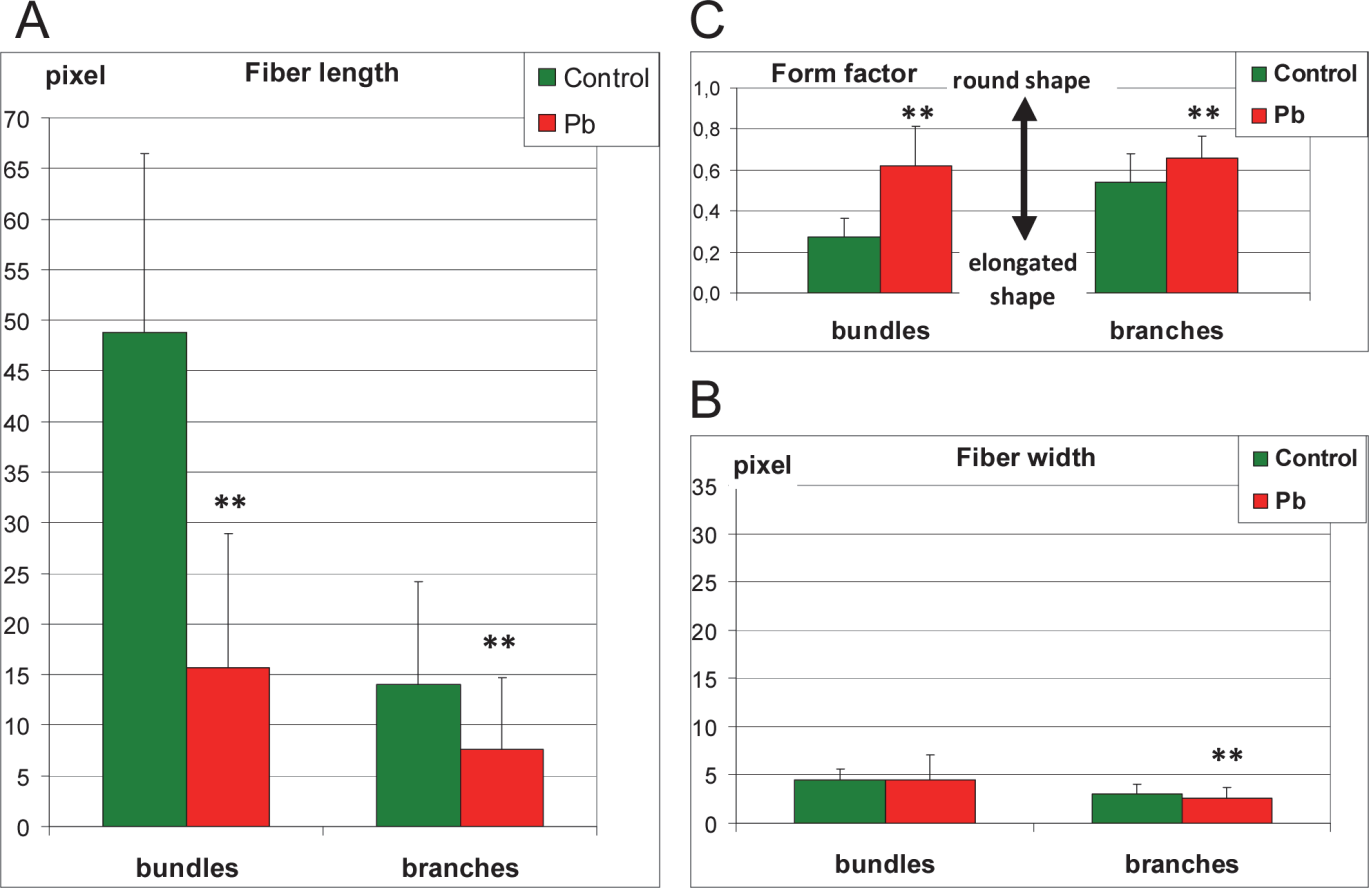
The analysis of morphology of actin filaments *Lemna trisulca* mesophyll cells. Determination of the differences in microfilament morphology was carried out based on the measurement of length (A—fiber length), thickness (B—fiber width) and shape (C—form factor) of microfilament bundles and their branches surrounding chloroplast in control plants and plants treated with lead in darkness. In cells treated with lead, disturbances in microfilaments morphology consisted of shortening (A) and rounding (C) both bundles and their branches. The width of microfilaments was lower in plants treated with lead (B) but only in the case of microfilaments branches. **p < 0.05

**2.4.1. Control plants**. Depending on the location of microfilaments in plant cells exposed to darkness, we distinguished a network of microfilaments surrounding chloroplasts, and other microfilaments (Fig. 7A and B). The former were formed long bundles running between plastids (Fig. 7A and B) and ramifying into short branches twisting around plastids in the form of structures resembling basket weaves (Fig. 7C). Among the microfilaments surrounding plastids, the longest bundles were also the thickest ones (Fig. 8B). Normally, all the fibers adhered to the surface of plastids. A network of microfilament bundles, which were not directly connected with chloroplasts was also seen in the thin layer of cytosol (Fig. 7B). Among them we identified long thick bundles, parallel to the cell surface, and their branches, which constituted a dense network of short and narrow bundles branching in various directions. Long bundles were often linked with the microfilament bundles surrounding chloroplasts.

**2.4.2. Lead**. In the fronds of plants treated with lead, no changes were recorded in comparison with control plants both in structure and in the thickness of microfilaments not connected with chloroplasts (Fig. 7G, compared with Fig. 7B). However, in the case of microfilaments surrounding chloroplasts, their images were only sporadically similar to those generated for control plants (Fig. 7D). In most cells deformations of the microfilament network structure were discovered. Apart from long bundles, linearly distributed microfilament fragments were also seen along the edges of chloroplasts (Fig. 7E). There were no microfilaments near some chloroplasts, or they were seen only locally (Fig. 7E), e.g. near one of the poles (Fig. 7G). This applied to both long and short (basket-forming) fibers. In individual cases, chloroplast clusters almost without any surrounding microfilaments were seen. In such places, thick, local clusters of actin were discovered near plastids (Fig. 7E). The quantitative analysis of morphology of microfilaments bundles and their branches surrounding the chloroplast showed their significant shortening and rounding in comparison to the microfilaments of control plants (Fig. 8A and C). The short microfilament branches twisting around chloroplasts were also generally thinner than in control plants (Fig. 8B) but in some cases they seemed thicker (Fig. 7F). The increased thickness was not accompanied by increased fluorescence intensity, as in some cases the intensity was even smaller than in control plants, and the actin filaments were more diffused.

### 2.5. Presence of hydrogen peroxide (in darkness)

**Fig 9:**
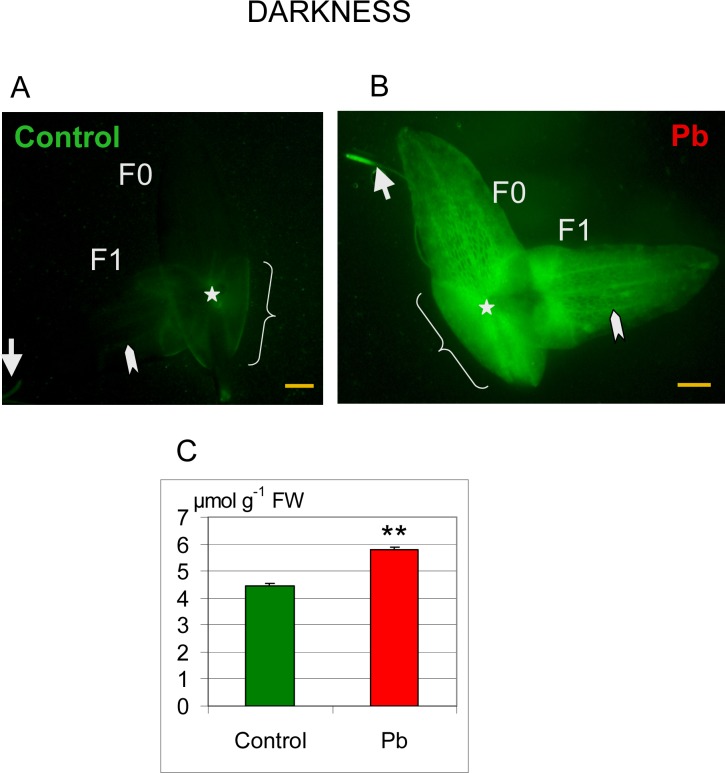
Distribution of hydrogen peroxide in *Lemna trisulca*. H_2_O_2_ localization by labeling with dichlorodihydrofluorescein diacetate (DCFH-DA; green fluorescence) in fronds (F0—mother fronds and F1—daughter fronds) and in the root of control plants (A) and plants treated with lead (B) in darkness. The control plants showed weak fluorescence in the area of the node (asterisk), sheaths (curly bracket), in the root tip (arrow) and in vascular bundles (arrowhead). Fluorescence intensity was higher in plants treated with lead (B) than in control plants (A). Additionally, under the influence of lead, hydrogen peroxide was generated in mesophyll cells of fronds (double arrow). (C) H_2_O_2_ content was also determined spectrometrically in control (Control) plants and plants treated with Pb. **p < 0.05; Scale bar = 1mm

A low fluorescence level reflecting a relatively low H_2_O_2_ content was detected in control plants in the area of the node and sheaths covering the newly formed fronds (Fig. 9A). Some fluorescence was also seen at the level of vascular bundles and in the root tip.

In contrast, the intensity of fluorescence significantly increased after treating plants with lead. The increase was distinct in the places showing low fluorescence in control plants (Fig. 9B), i.e. in the area of the node, sheaths, vascular bundles, and the root tip. Intense fluorescence appeared also in the places where it was absent in control plants: mesophyll cells of fronds and root cells between the root tip and basal part of root.

Microscopic findings concerning the increase in H_2_O_2_ content were confirmed by results of spectrometric measurements, as H_2_O_2_ content was significantly higher (1.3-fold) in plants treated with lead than in control plants (Fig. 9C).

### 2.6. Relationship between location of chloroplasts and lead content (in darkness)

**Fig 10:**
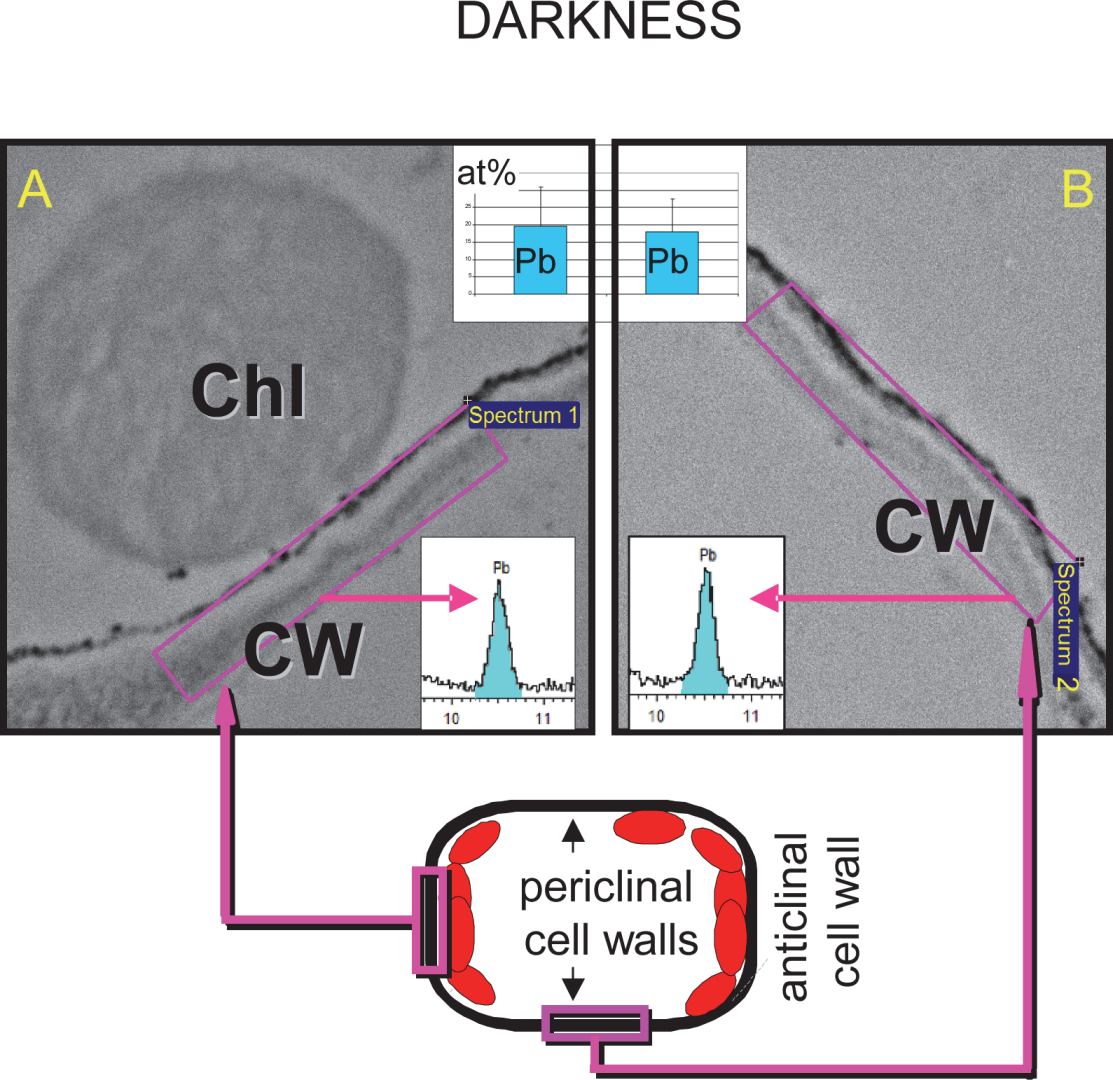
Colocalization of chloroplasts and Pb in *Lemna trisulca*. Lack of relationship between the location of chloroplasts and lead deposits in cell walls of plants treated with the metal in darkness. Lead content is shown as atomic percentage (at%, histograms) and as fragments of X-ray spectra (blue color marks the energy range corresponding to lead atoms) based on X-ray map microanalyses performed in areas of the same size (*n* = 30). Sample areas of such analyses in regions with chloroplasts (A) and without them (B) are shown as pink rectangles on images from transmission electron microscopy. Below, a diagram of a mesophyll cell section is presented, with marked sample areas of the analyses. Chl—chloroplast, CW—cell wall.

In order to determine whether chloroplast avoidance response to lead in darkness is attributable to keeping away from places of increased lead content, we compared the lead content of the areas where chloroplasts accumulated and in chloroplast-free places. X-ray microanalyses did not reveal any statistically significant differences in lead content (expressed as atomic concentration, at%) between these areas (Fig. 10). In the areas where chloroplasts were present, lead content amounted to about 19.6 at%, while in places where the organelles were absent, it was about 18.1 at% (the differences were statistically insignificant; Fig. 10). Calcium content was also determined in these areas. In areas of chloroplast aggregation it was slightly but not significantly higher (about 2.6 at%) than in the other places (about 1.8 at%).

## Discussion

Chloroplast movements are among the most important mechanisms used by plants to adapt to variable light conditions [4,5,17,18,19]. The movements may also be modified by other environmental factors [19,106]. In particular, lead ions are especially dangerous to the correct functioning of the photosynthetic apparatus [107,108,109]. Therefore, the toxicity of the metal in the context of chloroplast functioning in various light conditions should be determined—especially when lead is an additional stressor. For example, *Pisum sativum* L. plants growing in weak light (50 μmol m^-2^s^-1^) were more sensitive to lead treatment than those growing in strong light (600 μmol m^-2^s^-1^) [110], since they had a lower capacity for utilization of absorbed light. It was not clear whether this could be connected with differences in chloroplast exposure to light resulting from the impact of lead on the change of motor response of chloroplasts and their location in the cell.

### 3.1. Physiological aspects of Pb-induced avoidance-like chloroplast movements

Our results show that chloroplast movements should be taken into account while assessing the impact of lead on the photosynthetic apparatus of plants. We have established for the first time that lead ions caused significant changes in the distribution of chloroplasts in plants, using the mesophyll of the aquatic plant *Lemna trisulca*. Regardless of light conditions and additional treatments, about 20% more chloroplasts populated anticlinal cell walls of the lead-treated plants, in comparison with control plants. Thus the number of chloroplasts along periclinal walls in the face position automatically decreased. Only in strong white light, i.e. under conditions in which the profile position is a dominant chloroplast orientation, no differences between control plants and plants treated with lead were recorded. Differences caused by lead in chloroplast distribution in other conditions may affect the results of physiological tests. This applies in particular to the tests in which measuring methods depend on morphological features of plants, such as field measurements of chlorophyll content with the help of non-destructive chlorophyll meters based on the measurement of leaf transmittance, e.g. SPAD-502 [111]. For example, in *Nicotiana tabacum* leaves the relative chlorophyll level detected by the meter decreased as much as to 35% within a short time. This did not reflect the actual chlorophyll content, as chloroplasts only changed their orientation from the face to profile position. Failure to take into account changes in chloroplast orientation resulted in significant measurement errors [111]. The above example depicts consequences of changes in chloroplast orientation caused by differences in the strength of light the plants were exposed to. Our tests on *L*. *trisulca* show that lead also caused changes in chloroplast positioning, despite the same growing conditions. This fact should be taken into account in physiological tests checking the impact of lead on chloroplasts.

Our research revealed a particularly interesting change in the distribution of *L*. *trisulca* chloroplasts under the influence of lead in darkness. Despite the absence of light, the distribution of chloroplasts under the impact of lead resembled their distribution typical of avoidance response caused by strong light. In darkness, chloroplasts of control *L*. *trisulca* plants were typically distributed evenly along all the walls [29,47,51] (Fig. 2 A and B), while in the presence of lead most of them were located along anticlinal walls in the profile position, i.e. similarly as in strong light (Fig. 5C and D compared with Fig. 5 I-L). A question arises about the importance of the change in chloroplast distribution in darkness for the plant. It is not easy to find an answer, since little is known about the role of chloroplast distribution in darkness [13,18]. So far, the majority of studies on chloroplast movements focused on their functions in response to light, rather than in darkness. This is understandable in view of the key role the light plays in chloroplast function. It may also result from difficulties in the interpretation of chloroplast distribution in darkness, as there is no uniform model of chloroplast distribution in darkness for all plant species [19]. Although in strong light chloroplasts of most plants position themselves in a similar way, i.e. along anticlinal walls (parallel to the direction of light), the issue is more complex in the case of darkness. It is generally agreed that in the cells of embryophytes, chloroplasts are distributed evenly along all the walls in darkness. Such a distribution was indeed seen not only in the cells of *L*. *trisulca* mesophyll, but also in some flowering land plants, such as *Tradescantia* [4,11]; (Fig. 2 A and B). However, chloroplasts were located in darkness only along anticlinal walls in gametophyte cells of ferns (e.g. *Adiantum capillus-veneris*) and mosses (e.g. *Funaria hygrometrica*) [4,13]. As a result, their distribution was similar to that adopted in strong light. In *Arabidopsis thaliana* palisade cells, asymmetrical distribution of chloroplasts was recorded [15,16]—no chloroplasts were found along periclinal walls neighboring with epidermal cells, but they were present along the other anticlinal and periclinal walls (neighboring with intercellular spaces or other mesophyll cells), and their distribution depended on light conditions. For example, in plants grown in weak light, most chloroplasts were accumulated at the bottom of cells, i.e. the side further from the adaxial surface of the leaf in the face position [15]. According to the cited authors, such a distribution of chloroplasts in darkness (corresponding to accumulation response in weak light) enabled them to absorb an optimum amount of light energy immediately after they were exposed to weak light. Owing to the above, they did not need any additional energy necessary to move and adopt an optimum position in weak light [15]. We observed an opposite situation for *L*. *trisulca* chloroplasts treated with lead in darkness—here, most chloroplasts were located along anticlinal walls in the profile position. Therefore, a sudden exposure of the plants to weak light required more chloroplasts to change their orientation from the profile to face position (optimum in these conditions) than in the control group, thus requiring an increase in the energy input used by the cell to move chloroplasts. On the other hand, the lead-induced response of *L*. *trisulca* chloroplasts might be good for the plant in the presence of another stressor—strong light. As already pointed out, chloroplasts of most of embryophytes adopt the profile position in strong light, which protects photosynthetic apparatus from photodamage. For example, it was established that plants with mutations impairing chloroplast avoidance response were more susceptible to oxidative stress under excess light [25,26,27,63]. The above becomes particularly significant when other stressors—such as trace elements, which are a source of oxidative stress—are present in the environment with strong light [108,112]. Most chloroplasts of *L*. *trisulca* plants treated with lead in darkness would already have the safe profile position when suddenly exposed to strong light. Thus they would not have to change their orientation from the face to profile position (express avoidance), which would require not only energy but also time. Since lead slowed down the movement of *L*. *trisulca* chloroplasts in strong blue light (Fig. 6), the response would no doubt last longer. The chloroplasts would be exposed to the harmful activity of strong light for a longer time. Thus the reaction of *L*. *trisulca* chloroplasts to lead may have a defense function, which is advantageous when strong light—an additional stressor—appears.

The similarity of the response of chloroplasts to lead in darkness to their reaction to strong light may also be interpreted differently. Chloroplast avoidance is a directional response to strong light, where chloroplasts move away from areas exposed to excess light [4]. This happens not only when the entire tissue is exposed to strong light, but also when a cell is exposed locally [4,63]. A question arises: is the response of *L*. *trisulca* chloroplasts to lead also directional, i.e. do chloroplasts avoid the places in which the stressor (lead) is present? Using microanalysis we have shown that chloroplasts do not avoid places containing more lead. Therefore, the reaction caused by lead should rather be considered an “avoidance-like” response. Although the distribution of chloroplasts resembles the avoidance response to strong light, it is not a directional response dependent on the lead-rich places in *L*. *trisulca* cells.

Then another question arises: why is this response similar to strong light avoidance if no light is present? Looking for an answer, we must bear in mind that the role of chloroplast movement is not limited to adaptations to light. Tsuboi and Wada [13] believe that the position of chloroplasts in darkness may play an important role in the exchange of photosynthates between adjacent cells. Hence, e.g. in the prothallium of *A*. *capillus-veneris* , composed of only one layer of cells [113], chloroplasts were adjacent only to anticlinal walls of neighboring cells [13]. They were not seen along any walls exposed to the external environment. The cited authors suggest that accumulation of chloroplasts along a border between two cells may be conducive to the exchange of photosynthates between the cells [13]. The mesophyll of *L*. *trisulca* fronds is also composed of a single cell layer, but in contrast to the thallus cells of *A*. *capillus-veneris*, it is bordered by epidermal cells on both sides. In darkness, in control conditions, we recorded chloroplasts along all the walls, not only anticlinal ones. After administration of lead ions, the distribution of *L*. *trisulca* chloroplasts in darkness changed like in *A*. *capillus-veneris*. In these conditions, most chloroplasts adhered to anticlinal walls of the adjacent mesophyll cells, rather than to the epidermis. The question arises: may the change in chloroplast distribution result from lead-induced changes in the availability of substrates and photosynthates exchanged between cells? This cannot be excluded, since lead causes a disruption of numerous physiological processes, including CO_2_ assimilation and carbohydrate metabolism [112,114,115,116]. These processes are connected with the distribution of chloroplasts in cells. According to one hypothesis, plants may increase CO_2_ availability via a specific accumulation of chloroplasts along the walls neighboring with intercellular spaces. It is then probable that the efficiency of gas exchange increases as the length of CO_2_ diffusion pathway from intercellular spaces to chloroplast stroma gets shorter [117,118]. Yet we have not recorded any preferential accumulation of chloroplasts along cell walls neighboring with intercellular spaces in *L*. *trisulca*. Chloroplasts of control plants avoided places neighboring with intercellular spaces in weak light. In peripheral parts of cells, we recorded areas free from chloroplasts along anticlinal walls adjacent to intercellular spaces (Fig. 5 E and F—white arrow). This is in contrast to *Arabidopsis thaliana*, where chloroplasts accumulated around intercellular spaces in dim light [118,119,120]. The differences may result from a different structure and ecophysiology of *L*. *trisulca*. In the analyzed area of *L*. *trisulca* fronds, i.e. on their edges, mesophyll cells adhere to each other, creating a single layer of cells [4,100] (Figs. 2 and 5). Intercellular spaces located between epidermal walls and anticlinal walls of several mesophyll cells are small (S2 Fig.), in contrast to large intercellular spaces in the mesophyll of *A*. *thaliana*. In this way, the surface area per unit volume in *A*. *thaliana* mesophyll contributed to an increase in the efficiency of gas exchange [121]. In *L*. *trisulca*, an extensive gas exchange surface results from the presence of aerenchyma located only in the central part of fronds, in the node area [100]. This area was not covered by our microscopic examinations. In *L*. *trisulca* neither aerenchyma nor intercellular spaces are directly connected with the external environment. The totally submersed fronds have no stomata [100], which in land plants are the main structures facilitating gas exchange from ambient air to the intercellular space [122]. Gas diffusion from the water environment to individual plants representing this species takes place via a thin layer of cuticle, instead of stomata and substomatal cavities [100,123].

The shift of chloroplasts might also serve to increase the exchange of photosynthates between mesophyll cells [13] under lead-induced stress. As already pointed out, lead disturbs many metabolic processes, including transformations of carbohydrates [114,124,125]. Disturbed carbohydrate balance may affect the movements of chloroplasts [80,126]. Exogenous sucrose and glucose were shown to reduce chloroplast responses to strong and weak blue light in *L*. *trisulca*, with glucose being more inhibitory [80]. Sugars were suggested to modify the phototropin2-mediated signal transduction pathway [80]. Similarly to carbohydrates, lead decreased also the amplitude and kinetics of light-activated chloroplast responses in *L*. *trisulca* but, in contrast to carbohydrates [80], it hampered the avoidance more than the accumulation response (Fig. 6). Differences in response to carbohydrates and lead also included the impact on the motor apparatus—the actin cytoskeleton. In the case of carbohydrates, inhibition of chloroplast response was not due to a disturbed actin cytoskeleton of *A*. *thaliana* [81]. Our research showed that lead disturbed cytoskeleton structure in *L*. *trisulca* (Fig. 7). Further experiments are required to elucidate the role of sugar signaling in chloroplast responses to Pb.

### 3.2. Role of hydrogen peroxide and calcium in Pb-induced avoidance-like response

The response to lead may be connected with phot2-mediated responses. In *A*. *capillus-veneris* and *A*.*thaliana* phot2 was shown to be responsible not only for the avoidance response but also for proper dark positioning [37,45,63,127,128]. The unusual position in darkness induced by Pb in *L*. *trisulca* cells was similar to chloroplast avoidance response in strong light in control plants. This strategy appears to be characteristic not only of strong light or lead ions. An avoidance-like response was also recorded for mechanical stimuli [73] and low temperature [69,70,83]. Similar responses were also recorded after exogenous administration of important elements of signaling pathways—H_2_O_2_ [50] and calcium ions [47]. Can this similarity of chloroplast responses to various stressors result from joint mechanism(s) mediated by the same receptors and signaling pathways? The answer would help us understand plant defense reactions to various stressors.

In this context, the possible role of H_2_O_2_ seems to be particularly important, since increased production of reactive oxygen species (ROS) is among the main results of the impact of trace metals on plants. It normally leads to oxidative stress and, in consequence, to damage to biological membranes, changes in nucleic acids and photosynthetic pigments [93,108,112,125,129,130,131]. The ROS generated in the presence of metals may also act as signaling molecules [108]. H_2_O_2_ is an example of ROS whose content increased in the presence of lead ions. A similar effect was found in many species of land plants, including *Allium sativum* [132], *Brassica campestris* [125], *Lupinus luteus* [133], *Pisum sativum* [102], *Sedum alfredii* [134], *Talinum triangulare* [135], *Triticum aestivum* [136], and *Vicia faba* [137]. There is little information about aquatic plants in this context. We know, however, that lead stimulates the production of H_2_O_2_ also in aquatic plants, e.g. in macrophytes like *Najas indica* [138] and *Vallisneria natans* [98]. Our experiments with the small-sized macrophyte *L*. *trisulca* also confirm it. We have shown that in the fronds of the plants treated with lead (15 μM Pb^2+^) in darkness for 24 h, the content of H_2_O_2_ increased by more than 25% (Fig. 9). In the presence of stressors, H_2_O_2_ may therefore play a double role: in small concentrations it acts as an intracellular messenger triggering tolerance response, while in large concentrations, it may even lead to cell death, e.g. as a result of damage to biological membranes [139,140,141,142].

Strong light is among other stressors stimulating the production of H_2_O_2_ [142,143,144,145]. In experiments with *A*. *thaliana* the reactions were caused mostly by strong blue light [50], which induced the avoidance response in chloroplasts [29,30,31,32]. Those findings led us to hypothesize that H_2_O_2_ may be involved in chloroplast avoidance response. Zurzycki was the first to present a hypothesis on the participation of H_2_O_2_ in directional responses of chloroplasts [146]. As early as in 1972 he suggested that directional movements of chloroplasts may be chemotactic responses to local changes in concentration gradients of products of enzymatic reactions controlled by light, and he listed H_2_O_2_ among the substances able to cause movements of chloroplasts in this way [146]. Later research using *A*. *thaliana* showed that the 2 processes induced by strong blue light, i.e. chloroplast avoidance response and an increase in the concentration of H_2_O_2_ in mesophyll cells, may indeed be interrelated [50]. According to those authors, H_2_O_2_ probably plays the role of a second messenger molecule in chloroplast avoidance response in the presence of a stressor in the form of strong blue light.

Since the increased synthesis of H_2_O_2_ under the impact of various stressors is universal, a question arises: can any other stressors apart from strong light, e.g. trace metals, also trigger chloroplast avoidance response involving H_2_O_2_? We have decided to check whether lead ions influence directional movements of chloroplasts via the related increase in the level of H_2_O_2_. To eliminate the light factor, which might additionally influence the level of H_2_O_2_, we have carried out many of our experiments in darkness.

Lead concomitantly caused an increase in the concentration of H_2_O_2_ in *L*. *trisulca* (Fig. 9) and significantly changed the distribution of chloroplasts, bringing about an avoidance-like response. The response of *L*. *trisulca* chloroplasts to lead was partly related to the *A*. *thaliana* chloroplast response to exogenously administered H_2_O_2_ [50]. In strong blue light, H_2_O_2_ intensified the avoidance-like response in *A*. *thaliana*. In weak blue light, instead of an accumulation response, H_2_O_2_ triggered an avoidance response. The cited authors concluded that H_2_O_2_ intensified or induced chloroplast avoidance responses depending on the light intensity used. However, they did not record a similar phenomenon in plants kept in darkness [50]. In *L*. *trisulca* the induction of the avoidance response by H_2_O_2_ and lead took place in darkness. In the case of lead, a partial avoidance response was also recorded in weak light. In strong light, in the presence of lead, avoidance responses took place, which did not differ from typical responses of chloroplasts in such light conditions. On the other hand, photometric tests using strong blue light after a period of darkness showed a decreased amplitude and kinetics of avoidance response in the presence of lead in *L*. *trisulca*, in contrast to the situation recorded for *A*. *thaliana* after H_2_O_2_ treatment. Thus both factors, lead in *L*. *trisulca* and H_2_O_2_ in *A*. *thaliana*, induced chloroplast avoidance responses. The observed differences may result from different experimental conditions, such as duration of experiments.

Additional experiments with exogenously administered H_2_O_2_ and catalase confirmed the hypothesis that H_2_O_2_ may participate in chloroplast responses to lead in *L*. *trisulca* cells. Firstly, in darkness, *L*. *trisulca* showed an identical chloroplast response to exogenous H_2_O_2_ (both in terms of quantity and quality) with lead-induced avoidance response (Figs. 2 G and H, 3 and 4). Secondly, when catalase was added to the lead-containing medium in darkness, the extent of chloroplast avoidance response diminished in comparison with the response induced in the presence of lead or H_2_O_2_. As a result, an intermediate chloroplast position, between dark and profile positions, was recorded. Interestingly, when only catalase was added exogenously in darkness, the chloroplasts took an intermediate position between dark and face positions—typical of an accumulation response in weak light. A similar effect was described for *A*. *thaliana* cells after the administration of catalase [50]. This H_2_O_2_-specific antioxidant suppressed not only the generation of H_2_O_2_ induced by strong blue light, but also chloroplast movements imitating avoidance response [50]. The above examples show that H_2_O_2_ participates in chloroplast avoidance responses induced not only by strong blue light (in *A*. *thaliana*) [50], but also by lead (in *L*. *trisulca*). We cannot exclude that H_2_O_2_ plays a role of an intracellular messenger also in the case of lead. Further research is necessary, since the role of H_2_O_2_ as an intracellular messenger in chloroplast movements has not been fully determined yet. For example, some doubts concern experiments involving mannitol, which lowers the level of H_2_O_2_ but does not affect high-fluence blue-light-induced chloroplast avoidance movements [80].

We cannot exclude the possibility that other cell components also play the role of signals in chloroplast responses to lead. A potential candidate are calcium ions, which participate in chloroplast responses to blue light [46,53,147]. Additionally, inhibition of lead absorption by calcium is associated with competition between these 2 cations for calcium channels [148,149,150]. We have found that cells of *L*. *trisulca* fronds treated with lead in darkness have slightly increased the content of calcium in the proximity of chloroplasts than in areas free from the organelles when chloroplast avoidance-like response is induced by lead. A partial chloroplast avoidance response in darkness was also induced in *L*. *trisulca* fronds when they were treated with Ca^2+^ ions [46,47]. However, even when ionophores facilitating their introduction into cells were applied simultaneously with calcium ions, only 32% of the full strong blue light response was observed [47]. Our experiments with lead-treated plants showed a higher similarity of chloroplast response to lead to strong light avoidance response of chloroplasts of control plants, as indicated by a similar share of chloroplasts positioned along anticlinal walls (72.2% and 79.7%, respectively). In earlier experiments, 1 mM ethylene glycol tetraacetic acid (EGTA, for 18 h)—an antagonist of calcium homeostasis, which reduces the influx of external calcium—entirely stopped the movements of *L*. *trisulca* chloroplasts [47]. The treatment of *L*. *trisulca* with lead ions for 24 h lowered the amplitudes of chloroplast responses, but did not stop them (Fig. 6). The above discrepancies leave us with the question whether calcium ions participate in chloroplast responses to lead, and if so, to what extent. It is difficult to find an answer to the question insofar as the mechanism of the activity of calcium ions in chloroplast movements has not been explained yet. At least 2 roles have been discussed: Ca^2+^ ions transmit the light-generated signal downstream of the phosphoinositide kinases and/or control the motor apparatus [48,49,53,151]. The former mechanism is supported by hampering chloroplast responses in *L*. *trisulca* and *A*. *thaliana* by wortmannin—an inhibitor of phosphoinositide-3-kinase [46,47,48,53]. Notably, this inhibition took place without changes in the structure of the actin cytoskeleton [53]. However, disturbances in the Ca^2+^ homeostasis may also affect chloroplast movement through its influence on actin filament structure or motor molecule activity [48,53,151].

### 3.3. Actin cytoskeleton disturbances by lead

It was interesting to determine whether bivalent lead ions may also affect the positioning of chloroplasts by affecting the structure of the actin cytoskeleton. Our results indicate that it is highly probable, as we showed that lead changed the architecture of microfilaments in the mesophyll of *L*. *trisulca*. The observed structure of the actin cytoskeleton in control dark-adapted cells resembled the microfilament pattern recorded in cells of the land plants *A*. *thaliana* and *N*. *tabacum*. The pattern consisted of a meshwork of thin bundles around chloroplasts resembling a “compact basket”, attached to larger cortical actin cables [52,53,58]. The organization of the actin cytoskeleton in *L*. *trisulca* was different from that recorded in another flowering aquatic plant—*Vallisneria gigantea* [152]. No basket-like meshwork was seen in *V*. *gigantea* in darkness. Also, in *L*. *trisulca* in darkness we did not record any short actin filaments, called chloroplast actin (cp-actin) filaments, on the chloroplast envelope. These filaments were detected in *Arabidopsis* expressing a green fluorescent protein (GFP)-mouse talin fusion protein [59].

The changes in the pattern of the actin cytoskeleton, resulting from the impact of lead in *L*. *trisulca*, covered only the network of microfilaments directly surrounding the chloroplasts (Fig. 7). However, they may have been sufficient to result in changes in chloroplast positioning. It is suggested that actin baskets and their interactions with the cortical actin cytoskeleton play a key role in chloroplast positioning in higher land plants [52,53,58]. Among the lead-activated changes affecting *L*. *trisulca* microfilaments, we recorded division into shorter stretches (fragmentation), and in some cases their local or even total disappearance next to chloroplasts. Instead, local actin clusters appeared, which were often located near a pole of the chloroplast (Fig. 7 E and G). As can be seen, the alterations were not of the type recorded in the light inducing chloroplast movements in *A*. *thaliana* and *N*. *tabacum*. The only difference in the image of actin bundles after strong light irradiation was their widening/diffusion, while the architecture of microfilament networks remained unchanged [52,53]. Lead-induced changes in the actin cytoskeleton in *L*. *trisulca* resembled disturbances similar to those induced by stronger stressors. Fragmentation of the actin cytoskeleton and/or formation of its clusters (punctate foci) were recorded under the influence of artificially-induced changes in the concentration of cytosolic free Ca^2+^ [53,153], low temperatures [154], and ions of other trace metals, e.g. Al^3+^ and Cd^2+^ [86,155]. Considerable disturbances in the structure of the actin cytoskeleton were also recorded as a result of the activity of latrunculin B or EGTA, which disorganized the distribution of chloroplasts in the cell [53,58,119]. One of the results of the impact of these factors was the formation of chloroplast clusters, which normally happened in very strong light [12,46]. Although such chloroplast clusters were seen in *L*. *trisulca* in the presence of lead, they appeared only sporadically. Additionally, the positioning of chloroplasts in darkness after treatment with lead was not accidental—it was related to their orderly pattern in the strongly irradiated cells of control plants. Why then did the chloroplasts react with this specific avoidance-like response to lead, despite clear changes in the structure of the actin cytoskeleton? According to Oikawa et al. [156], the distribution of chloroplasts along the anticlinal plasma membrane is the default position for chloroplasts when their relocation becomes defective, e.g. as a result of the cytoskeleton losing its motor functions. That conclusion was drawn on the basis of their research on the transgenic *A*. *thaliana* plants expressing truncated chloroplast outer envelope protein (CHUP1) in a *chup1* mutant. Their tests showed that chloroplasts in the transgenic lines lacking an actin-binding region of CHUP1, regardless of light conditions (also in darkness), were distributed only along anticlinal walls. Those authors also demonstrated that the coiled-coil region of CHUP1 (N-C region) was responsible for anchoring chloroplasts firmly in the plasma membrane. Since the protein(s) interacting with the coiled-coil region of CHUP1 exist only in the anticlinal plasma membrane in the N-C line plants, chloroplasts may be anchored only along anticlinal walls [156]. By analogy, although lead disturbed motor responses of chloroplasts (e.g. by disturbing connections between chloroplasts and the actin cytoskeleton) in *L*. *trisulca*, chloroplasts maintained their ability to get anchored in the cell membrane.

## Conclusions

Our results confirm the hypothesis that changes in motor responses of chloroplasts of *L*. *trisulca*, leading to their redistribution in the cell, are one of the mechanisms of the impact of lead on the organelles. We have shown for the first time that the changes involved a redistribution of chloroplasts towards anticlinal walls. The biological significance of lead-induced chloroplast responses remains unclear. The possible interpretations of this phenomenon, presented by us, can be grouped into 3 basic categories: physiological, pathological, and cross-talk. Firstly, the avoidance-like response of chloroplasts may be a form of adjustment of the photosynthetic apparatus to lead-induced physiological changes in the availability of CO_2_ or photosynthates. On the other hand, we did not confirm the hypothesis that similarly to the physiologically important avoidance of strong light, the change in positioning of chloroplasts in the presence of lead is also a directional avoidance of the places where lead was accumulated. We cannot exclude, therefore, that this reaction is pathological. This seems to be confirmed by our results concerning the impact of lead on the actin cytoskeleton, since we have shown for the first time the disturbances in the structure of the chloroplast-related actin cytoskeleton in the presence of lead. The disorders in the actin cytoskeleton by lead may, in turn, be the reason of the dysfunctions of blue-light-induced chloroplast movements. Especially strong reduction of chloroplast responses to lead has been shown in the case of strong blue light. Such a strong reaction of chloroplasts to the simultaneous presence of 2 stress factors—lead and strong light—may indicate a cross-talk. In addition, our results suggest that H_2_O_2_, which is produced in response to a variety of stresses (including bright light and heavy metals) can be involved in the avoidance-like movement of chloroplasts induced by lead. The participation of reactive oxygen in Pb-induced avoidance-like response is a premise to consider in the future the phenomenon of the reaction of chloroplasts to lead in terms of cross-talk. A comprehensive explanation of the mechanisms of the observed chloroplast responses to lead requires further research involving the subsequent stages of the responses, i.e. the reception of signals (does it involve phot2?), signal transduction (do Ca^2+^ and/or H_2_O_2_ ions participate in it and, if yes, to what extent?), and changes in the motor system area (e.g. those concerning motor proteins and proteins participating in the anchoring of chloroplasts). Another question to be solved is to what extent the lead-activated changes in chloroplast positioning are disturbances, and to what extent they are defense reactions.

## Supporting Information

S1 Fig Scheme of micrograph reconstructions used in Figs. 2 and 5. The micrographs show the distribution of chloroplasts in mesophyll cells of *L*. *trisulca* recorded in a confocal microscope on the basis of autofluorescence of chloroplasts. The larger micrographs illustrate 3-dimensional (3D) reconstructions of frond fragments, obtained on the basis of a series of optical sections adjacent to the surface of fronds. These reconstructions include chloroplasts of a single layer of mesophyll cells, although in some places tiny epidermal chloroplasts are also visible. To emphasize the depth of micrographs, a colour scale was applied (warm colours represent chloroplasts located closer to the observer, while cold colours denote the more remote ones). In smaller micrographs, the arrangement of chloroplasts (red autofluorescence) was presented in cross sections of cells, obtained by a single optical section. The white rectangle shows schematically the outline of the cross section of one cell.
(TIF)

S2 Fig Intercellular space in mesophyll of *Lemna trisulca*. Transmission electron micrograph: fragments of frond cells (region of single layer of mesophyll cells) in control plant. Labels: EP—epidermis, MC—mesophyll cell, IS—intercellular space, Chl—chloroplast, CW—cell wall, N—nucleus. Scale bar = 2μm
(TIF)
