# Supplementary figures and images for: Pb-Induced Avoidance-Like Chloroplast Movements in Fronds of Lemna trisulca L
Source: PLoS One. 2015 Feb 3;10(2):e0116757. doi: 10.1371/journal.pone.0116757 (PMC4315572; doi:10.1371/journal.pone.0116757)

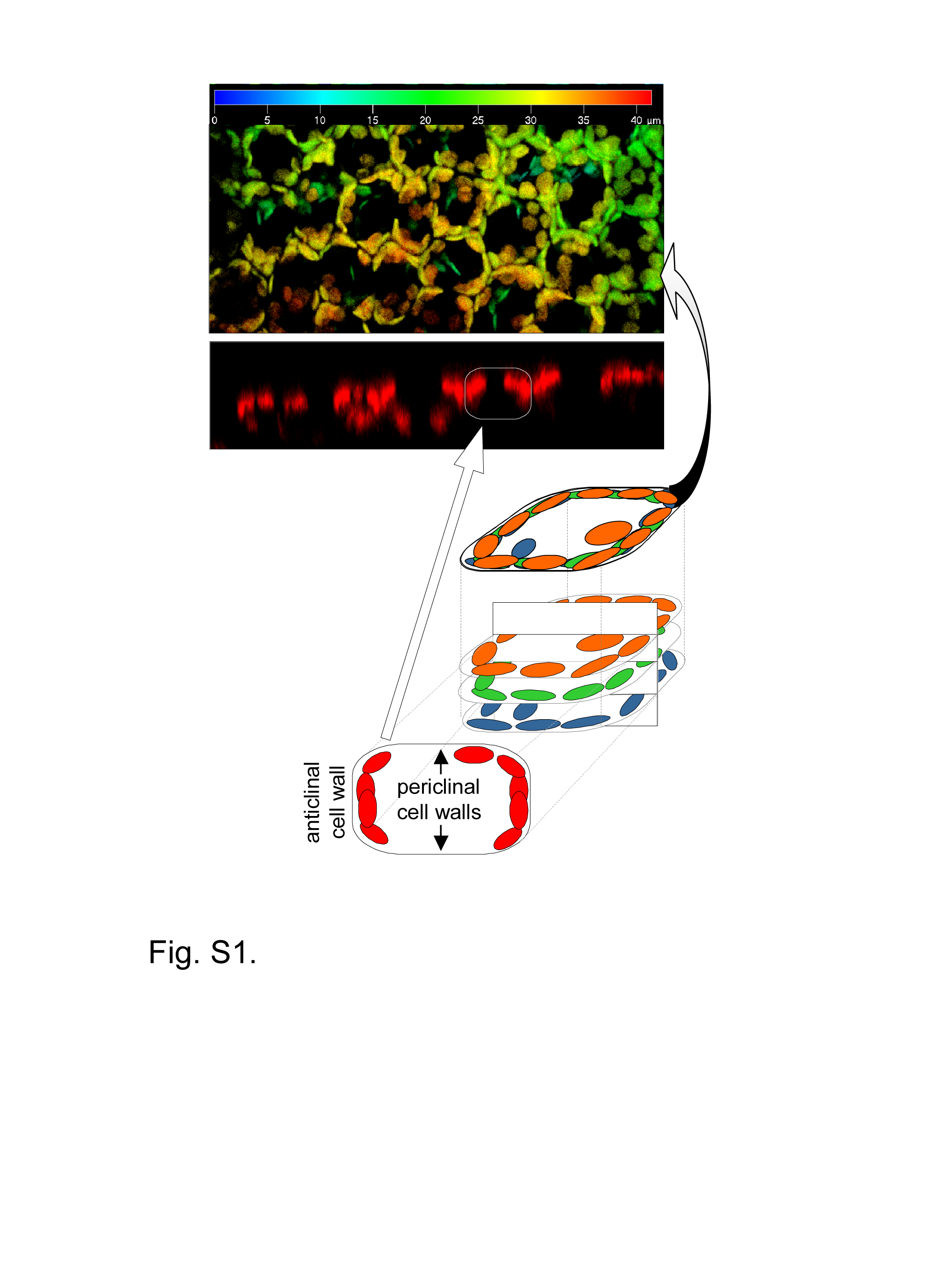

Supplement: S1 Fig — The micrographs show the distribution of chloroplasts in mesophyll cells of L. trisulca recorded in a confocal microscope on the basis of autofluorescence of chloroplasts. The larger micrographs illustrate 3-dimensional (3D) reconstructions of frond fragments, obtained on the basis of a series of optical sections adjacent to the surface of fronds. These reconstructions include chloroplasts of a single layer of mesophyll cells, although in some places tiny epidermal chloroplasts are also visible. To emphasize the depth of micrographs, a colour scale was applied (warm colours represent chloroplasts located closer to the observer, while cold colours denote the more remote ones). In smaller micrographs, the arrangement of chloroplasts (red autofluorescence) was presented in cross sections of cells, obtained by a single optical section. The white rectangle shows schematically the outline of the cross section of one cell. (TIF) [file pone.0116757.s001.tif]

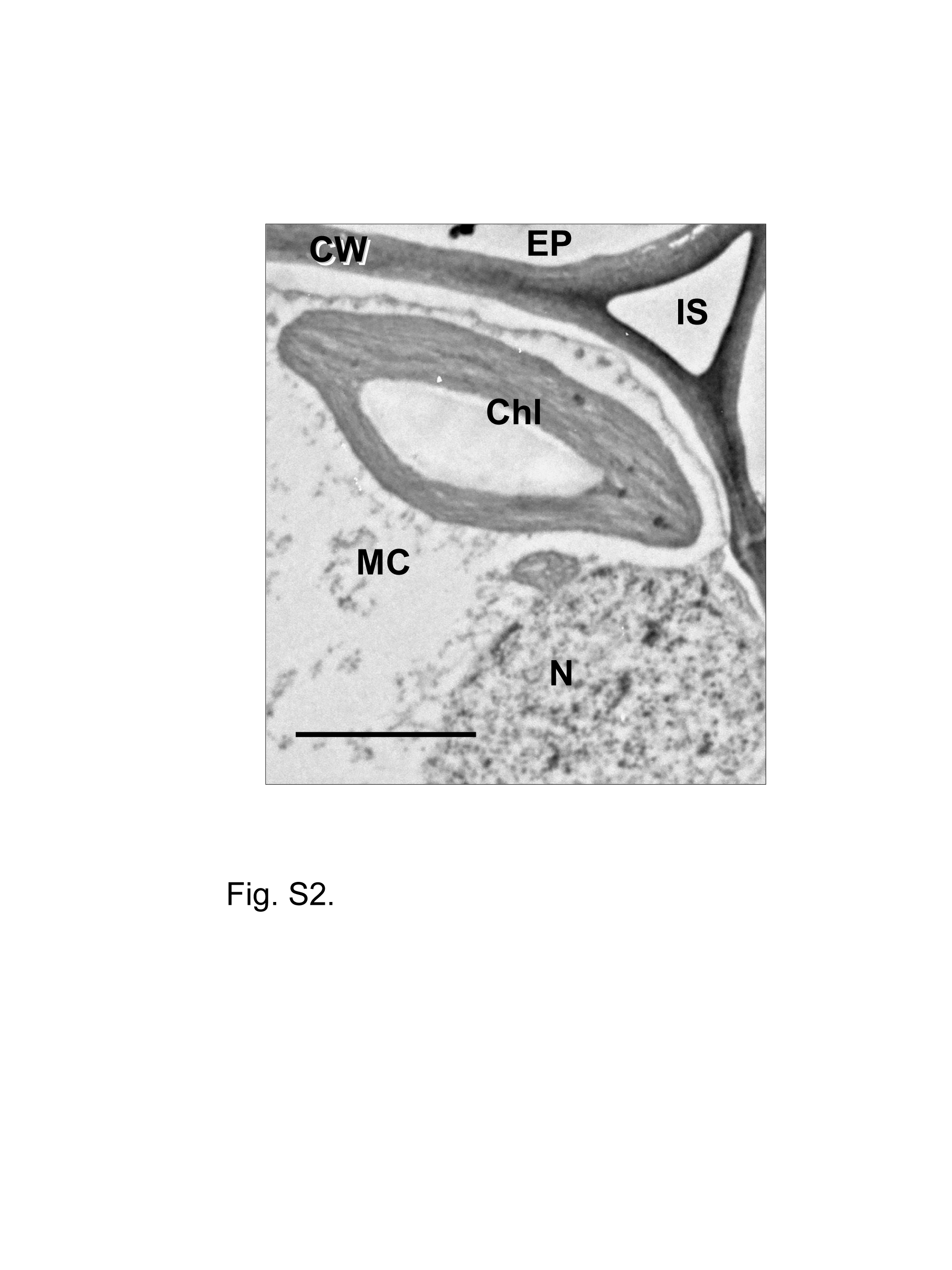

Supplement: S2 Fig — Transmission electron micrograph: fragments of frond cells (region of single layer of mesophyll cells) in control plant. Labels: EP—epidermis, MC—mesophyll cell, IS—intercellular space, Chl—chloroplast, CW—cell wall, N—nucleus. Scale bar = 2μm (TIF) [file pone.0116757.s002.tif]
